# Quantum coherent energy transport in the Fenna–Matthews–Olson complex at low temperature

**DOI:** 10.1073/pnas.2212630119

**Published:** 2022-11-28

**Authors:** Hong-Guang Duan, Ajay Jha, Lipeng Chen, Vandana Tiwari, Richard J. Cogdell, Khuram Ashraf, Valentyn I. Prokhorenko, Michael Thorwart, R. J. Dwayne Miller

**Affiliations:** ^a^Department of Physics, School of Physical Science and Technology, Ningbo University, Ningbo 315211, People’s Republic of China; ^b^Max Planck Institute for the Structure and Dynamics of Matter, 22761 Hamburg, Germany; ^c^I. Institut für Theoretische Physik, Universität Hamburg, 22607 Hamburg, Germany; ^d^The Hamburg Center for Ultrafast Imaging, 22761 Hamburg, Germany; ^e^The Rosalind Franklin Institute, Rutherford Appleton Laboratory, Harwell Campus, Didcot, Oxfordshire OX11 OFA, United Kingdom; ^f^Research Complex at Harwell, Rutherford Appleton Laboratory, Didcot OX11 0QX, United Kingdom; ^g^Zhejiang Laboratory, Hangzhou 311100, P.R. China; ^h^Department of Chemistry, Technische Universität München, 85747 Garching, Germany; ^i^Department of Chemistry, Universität, 20146 Hamburg, Germany; ^j^European XFEL GmbH, 22869 Schenefeld, Germany; ^k^School of Molecular Biosciences, University of Glasgow, Glasgow G12 8QQ, United Kingdom; ^l^Department of Chemistry, University of Toronto, ON, Canada M5S 3H6; ^m^Department of Physics, University of Toronto, ON, Canada M5S 3H6

**Keywords:** energy transfer, two-dimensional spectroscopy, excitonic coupling, coherent transport

## Abstract

In the primary step of natural light harvesting, the solar photon energy is captured in a photoexcited electron–hole pair, or an exciton, in chlorophyll. Its conversion to chemical potential occurs in the special pair reaction center, which is reached by downhill ultrafast excited-state energy transport through a network of chromophores. Being inherently quantum, transport could in principle occur via a matter wave, with vast implications for efficiency. How long a matter wave remains coherent is determined by the intensity by which the exciton is disturbed by the noisy biological environment. The stronger this is, the stronger the electronic coupling between chromophores must be to overcome the fluctuations and phase shifts. The current consensus is that under physiological conditions, quantum coherence vanishes on the 10-fs time scale, rendering it irrelevant for the observed picosecond transfer. Yet, at low-enough temperature, quantum coherence should in principle be present. Here, we reveal the onset of longer-lived electronic coherence at extremely low temperatures of ∼20 K. Using two-dimensional electronic spectroscopy, we determine the exciton coherence times in the Fenna–Matthew–Olson complex over an extensive temperature range. At 20 K, coherence persists out to 200 fs (close to the antenna) and marginally up to 500 fs at the reaction center. It decays markedly faster with modest increases in temperature to become irrelevant above 150 K. At low temperature, the fragile electronic coherence can be separated from the robust vibrational coherence, using a rigorous theoretical analysis. We believe that by this generic principle, light harvesting becomes robust against otherwise fragile quantum effects.

## Significance

We use ultrafast two-dimensional optical spectroscopy to study the primary steps in the energy transport in photosynthesis using the Fenna–Matthews–Olson complex as a model. By varying temperature to modify the intensity of decohering fluctuations, we determine the onset of quantum coherence effects at an ultralow temperature of 20 K. A theoretical analysis shows that this electronic quantum coherence can be separated from the pronounced vibrational coherences accompanying energy transport. We provide a complete picture of the lifetimes of electronic and vibrational quantum coherences and show that energy transport occurs under strong system–bath interactions. Electronic coherence can in principle sustain energy transfer at 20 K but rapidly fades away with increasing temperatures, becoming irrelevant under physiological conditions.

The question how biological function emerges from the atomic constituents of matter has intrigued scientists since the early days of quantum mechanics (1, 2). Encouraged by the success of quantum theory to describe matter, its pioneers rapidly explored extending it to the world of chemistry and biology in the early 20th century. It was only in recent times when modern ultrafast spectroscopic tools became available that the search for nontrivial quantum effects in the primary steps of biological processes was made possible, which led to the prospect for the foundation of the field of quantum biology (3–6). Recent experimental results obtained for the well-characterized Fenna–Mathews–Olson (FMO) protein complex have been interpreted as evidence of long-lived electronic quantum coherence in the primary steps of the energy transfer (7–10). A functional role of long-lived quantum coherence was proposed in that it would speed up the transfer of excitation energy under ambient conditions (11). The reports of long-lived coherences were in contradiction with earlier pump–probe studies (12) of the FMO complex at 19 K that found the electronic dephasing and modulations decayed on the order of 140–180 fs. This observable is complicated by possible inhomogeneous broadening that left the long-lived electronic coherences observed in two-dimensional (2D) spectra an open issue (7–10).

These works triggered tremendous interest in different fields, ranging from quantum chemistry to quantum information science. A key parameter is the strength of the coupling of the exciton system to environmental fluctuations, which is related to the reorganization energy. For a conceptual understanding, initial theoretical analysis was built upon the choice of a rather small reorganization energy of 35 cm^−1^ to fit the reported long lifetime of the electronic coherence (11). Yet, even with this small value, Shi and other researchers found a shorter lifetime for the expected electronic coherence from more advanced calculations of the experimental 2D electronic spectra (13–16). Moreover, it was shown that optimal energy transfer in the FMO complex in a thermal environment could also be achieved on the basis of a purely incoherent hopping on a downhill ladder structure (17, 18). The interpretation of the long lifetime of the electronic coherence was further questioned by numerically exact results

obtained from the quasiadiabatic propagator path integral method with an experimentally determined spectral density with a considerably larger reorganization energy (19–21). Coker et al. and Kleinekathöfer et al. have calculated site-dependent reorganization energies with refined atomic details by advanced molecular dynamics simulations (22, 23). They found significantly larger values of the reorganization energies in the range of 150–200 cm^−1^. With this disagreement, we have revisited the energy transfer of the FMO complex at room temperature experimentally (24) using 2D electronic spectroscopy to extract the electronic coherence time scales. Instead of a long-lived electronic coherence, the experiment, after having passed a self-consistency verification, yielded a considerably shorter coherence lifetime of 60 fs. This observed timescale for decoherence excludes any functional role for the coherent energy transfer in the FMO complex, which occurs on the time scale of several picoseconds at room temperature.

Another potential key role for the electronic coherence is played by the pigment–protein host molecular vibrations (25–28). In contrast to electronic coherence, the pigment-localized typically last for picoseconds but are not expected to enhance energy transfer in general. Yet, Plenio et al. have suggested the concept of vibrationally enhanced electronic (29). They reported that in a vibronic model dimer, electronic quantum coherence may be resonantly enhanced by long-lasting vibrational coherence (30). Instead of an enhancement, Tiwari et al. alternatively suggested that nonadiabatic electronic–vibrational mixing may resonantly enhance the amplitude of particular, delocalized anticorrelated vibrational modes in the electronic ground state (31). While in principle, this mechanism is also possible in the presence of weak electronic dephasing (32), realistic values of the strengths of the electronic and vibrational dampings lead to complete suppression of this mechanism (33). Electronic–vibrational mixing was also examined in a simple dimer model (34), but the subsequent theoretical calculations show no evidence of an enhancement of the electronic coherence (35). More recently, the coherent exciton transfer in the FMO complex at 77 K has been revisited by Zigmantas et al. (36). The long-lived oscillations have been carefully assigned to the vibrational coherence in the electronic ground state. Due to the strong dissipation, the lifetime of the electronic coherence was too short to be precisely determined even at 77 K. Temperature-dependent difference fluorescence line-narrowing (FLN) spectroscopy (37) has also revealed a roughly three-fold increase of the electronic damping and constant vibronic couplings in the available temperature range from 4.5 to 70 K. Despite the extensive work on this problem, a complete picture of the electronic coherence and its role in the electronic–vibrational mixing for the energy transfer in the FMO complex is still elusive.

Here, we study the energy transfer process in the FMO complex with the explicit aim to observe clear evidence for the onset of electronic coherence effects in energy transport by going to low temperature. To this end, we measure the 2D electronic spectra of the FMO complex in the regime of very low temperature. Specifically, we examine the electronic dephasing by directly measuring the antidiagonal bandwidth of the main peaks, along with the decays in the cross-peaks related to the interexciton coupling. It is only at very low temperatures (20 K) that the amplitudes and decays in these electronic coherence signatures become comparable to the energy transfer times. We provide a comprehensive analysis by a global fitting approach, and the subsequent Tukey window Fourier transform allows us to disentangle the electronic coherence from vibrational coherence. Based on these analyses, we uncover that the longest-lived electronic coherence is observable with lifetimes up to 500 fs between the two excitons closest to the reaction center side. Due to the downhill energy transfer, the electronic coherence of the two higher-energy excitons close to the antenna side exhibits a much faster decay with a lifetime < 60 fs. We furthermore measure the coherent energy transfer over an extensive temperature range. Based on these temperature-dependent measurements, we are able to construct a unifying exciton model to capture the coherent energy transfer in the FMO complex. Moreover, we investigate the temperature-dependent non-Markovianity of the transfer dynamics to show that the bath fluctuations are uncorrelated even at low temperatures. By this unprecedented combination of experimental and theoretical efforts, we are able to provide a complete picture of quantum coherent effects in the FMO complex over the entire regime from high to low temperatures in one experiment and one theoretical model. Due to the generic structure of the FMO protein, we expect our observations to be extended to other more complicated photosynthetic protein complexes and even photovoltaic devices (38).

## Results

**Fig. 1.**
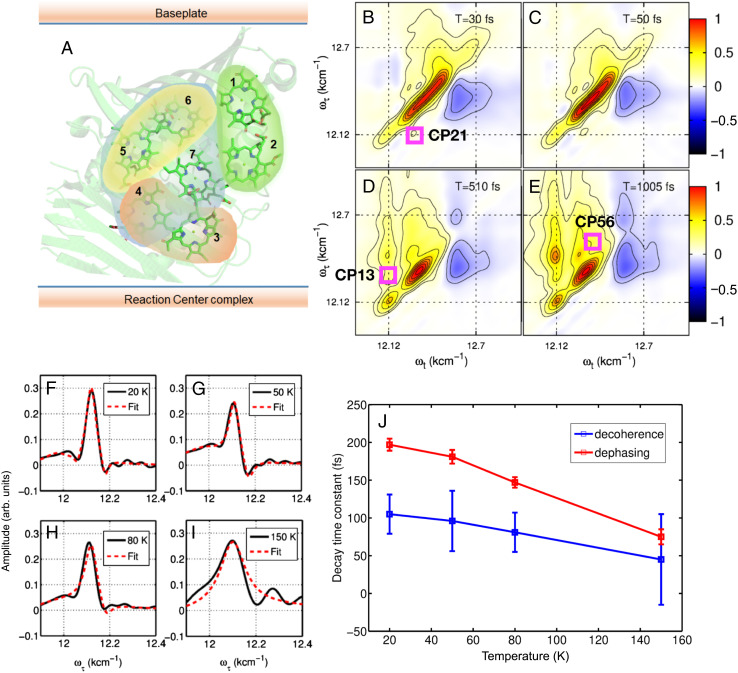
(*A*) Structural arrangement of the pigments in the FMO protein complex. (*B*-*E*) Real parts of measured 2D electronic spectra of the FMO complex at 20 K for waiting times of 30, 50, 510, and 1,005 fs, respectively. Here, $\omega_{\tau }$ is the excitation, and $\omega_{t}$ is the probing frequency. The photoinduced changes in the transmission caused by the excited-state absorption and the ground-state bleach are shown by the red and blue peaks, respectively. Black dashed lines indicate the frequency range between 12,120 and 12,700 cm^-1^ where the exciton states of the FMO complex are situated.} (*F*-*I*) Antidiagonal bandwidths of the diagonal peak at ($\omega_{\tau }$, $\omega_{t}$) = (12,120, 12,120) cm^-1^ for temperatures of 20, 50, 80, and 150 K. The decay times of the electronic dephasing between the ground and excited states are obtained by fitting to a Lorentzian line shape (red dashed line). We find 197±8, 181±9, 147±7, and 75±10 fs, respectively, which are marked as “dephasing” in (*J*). For comparisons, we also show decay time constants of the electronic coherence between 1 and 2 (marked as “decoherence”) obtained from Fig. 3.

The solution of the FMO protein complex is prepared in a homebuilt sample cell and mounted in a cryostat (Oxford Instrument). More details of the sample preparation are given in the *Materials and Methods*. Fig. 1*A* depicts the structural arrangement of the bacteriochlorophyll a (Bchla) chromophores embedded in the protein matrix (data from 3ENI.pdb). The measured absorption spectrum of the FMO complex at 80 K and the laser spectrum used in this study are shown in *SI Appendix*.

### Two-Dimensional Electronic Spectroscopy

We measure the 2D electronic spectra of the FMO complex in the temperature range from 20 to 150 K, which corresponds to bath thermal energies of 14–104 cm^−1^. The details of the 2D spectrometer are given in the *Materials and Methods*. In Fig. 1*B*–*E*, we show the real parts of the 2D electronic spectra at 20 K for selected waiting times *T* = 30, 50, 510, and 1,005 fs. Throughout this work in all 2D spectra, *ω*_*τ*_ and *ω*_*t*_ correspond to excitation and probing frequencies, respectively. The peaks with positive and negative amplitudes represent the excitation transitions of the ground-state bleach (GSB) and the excited-state absorption (ESA), respectively. The exciton states in the FMO complex are located in the frequency range from 12,120 to 12,700 cm^−1^, which is marked by black dashed lines. As shown in Fig. 1*B*, one readily observes that the main peaks stretch dramatically along the diagonal at *T* = 30 fs, illustrating a strong inhomogeneous broadening. In addition, one off-diagonal feature corresponding to the ESA is located at (*ω*_*τ*_, *ω*_*t*_) = (12,300, 12,580) cm^−1^. Increasing the waiting time to *T* = 50 fs (see Fig. 1*C*), the 2D spectrum does not change too much, except that the elongation of the main diagonal peaks is slightly reduced as compared to that at *T* = 30 fs. Upon further increasing the waiting time to *T* = 510 fs, the elongation of the main peaks along the diagonal is found to be dramatically reduced as illustrated in Fig. 1*D*. Moreover, the main peaks of the higher exciton states are replaced by one peak with ESA features. A new cross-peak emerges at (*ω*_*τ*_, *ω*_*t*_) = (12,340, 12,120) cm^−1^, which provides evidence of the downhill energy transfer from higher exciton states to the lowest ones. Its amplitude is further increased at *T* = 1,005 fs as displayed in Fig. 1*E*. Besides the main and cross-peaks in the frequency range from 12,120 to 12,700 cm^−1^, more cross-peaks emerge on the upper-left side of the 2D electronic spectrum at *T* = 1,005 fs, demonstrating the vibrational progression in the FMO complex (see detailed discussions in ref. (35)). To examine the lifetime of the electronic dephasing, we analyze the antidiagonal bandwidth of the lowest exciton peak at (*ω*_*τ*_, *ω*_*t*_) = (12,120, 12,120) cm^−1^ for *T* = 30 fs. To this end, we fit the antidiagonal bandwidth by Lorentzian line shapes according to the Redfield theory, which provides a straightforward way to estimate the lifetime of the optical dephasing. More details of the fitting procedure are described in *SI Appendix*. The antidiagonal bandwidths of the lowest exciton peak and the corresponding fitted curves at temperatures 20, 50, 80, and 150 K are shown in Fig. 1*F*–*I*. The extracted lifetimes of the electronic dephasing at temperatures 20, 50, 80, and 150 K are 197 ± 8, 181 ± 9, 147 ± 7, and 75 ± 10 fs, respectively, as marked by “dephasing” in Fig. 1*J*. We note that we operate in the temperature region of 20–150 K, which corresponds to bath thermal energies of 14–104 cm^−1^. From the FMO Hamiltonian in the site basis given in *SI Appendix*, Eq. S6, we see that the electronic couplings range from around 10–106 cm^−1^. Hence, the regime of low temperature, where the bath thermal energy is much smaller than all the electronic couplings, is not completely reached even at 20 K, and the dynamics live in the intermediate temperatureregime.

### Coherent Dynamics and Energy Transfer

**Fig. 2.**
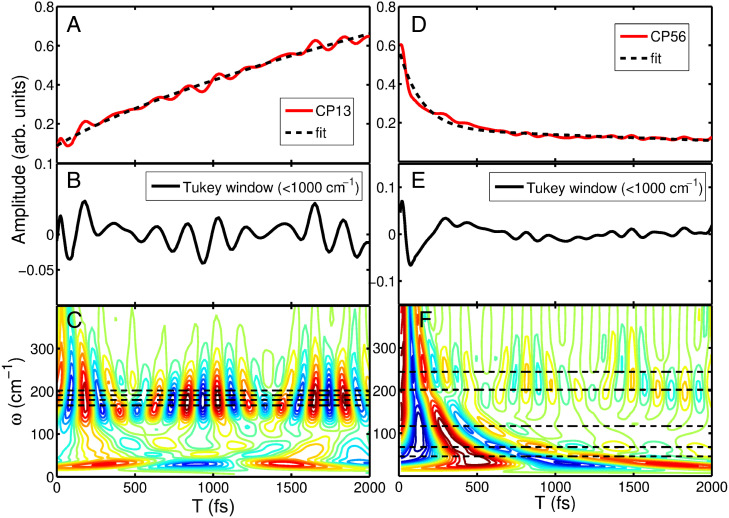
(*A*) Kinetics (red line) and fitted trace (black dashed line) of the cross-peak measured at ($\omega_{\tau }$, $\omega_{t}$) = (12,340, 12,120) cm^-1^. (*B*) The traces (black solid line) after removing high-frequency noise by a Tukey window Fourier analysis. (*C*) Wavelet spectrum of the time evolution of the vibrational modes with the frequencies at 167, 180, 191, and 202 cm^-1^, which result in the observed oscillatory resonant beating in the wavelet map. This beating is most readily observable as the modulated spectral intensity and associated maxima at waiting times of 1,000 and 1,700 fs. (*D*) Kinetics (red line) and fitted trace (black dashed line) of the cross-peak at ($\omega_{\tau }$, $\omega_{t}$) = (12,570, 12,480) cm^-1^. (*E*) Traces after Tukey window Fourier treatment (black solid line). (*F*) Wavelet spectrum with the coherent dynamics of the vibrational modes at 46, 68, 117, 202, and 243 cm^-1^.

To examine the coherent dynamics in the 2D spectra, we extract the amplitudes of the cross-peaks at different waiting times. In Fig. 2*A*, the trace (red line) represents the time evolution of the amplitude of the cross-peak at (*ω*_*τ*_, *ω*_*t*_) = (12,340, 12,120) cm^−1^ (marked as “CP13” in Fig. 1*D*). The underlying kinetics (black dashed line) is fitted by an exponential function, and the resulting residuals are shown as a black solid line in Fig. 2*B*. The raw data of the oscillations are further purified by a Fourier filter with a Tukey window (< 1,000 cm^−1^) in Fig. 2*B*. With this refined trace, we retrieve the coherent dynamics by performing a wavelet analysis. The details of the Tukey window Fourier transform and the wavelet analysis are given in *SI Appendix*. The time evolution of the cross-peak coherence is shown in Fig. 2*C*. We are able to resolve oscillations with frequencies in the range from 160 to 200 cm^−1^. More specifically, we identified four modes at 167, 180, 191, and 202 cm^−1^ in the electronic ground state, which are highlighted by black dashed lines in Fig. 2*C*. It should be noted that these mode frequencies are in excellent agreement with those obtained by FLN studies (39). Since the frequencies lie close by, we observe clear evidence of beating of vibrational oscillations at waiting times between 1,000 and 1,700 fs. Our analysis clearly reveals that the beatings originate from the resonant enhancement of individual vibrational modes at close-by frequencies in the electronic ground state. These vibrational beatings have been incorrectly assigned to the enhancement of electronic coherence by long-lasting vibrational coherences. Moreover, we show the traces of the cross-peak amplitudes (red solid line) at (*ω*_*τ*_, *ω*_*t*_) = (12,570, 12,480) cm^−1^ (marked as CP56 in Fig. 1*E*) and the corresponding fit (black dashed line) in Fig. 2*D*. The polished residuals (black solid line) obtained by the Tukey window Fourier transform are displayed in Fig. 2*E*. By further performing the wavelet analysis of the residuals, one obtains the coherent with resolved modes (marked as black dashed lines) in Fig. 2*F*. We found five vibrational modes with frequencies of 46, 68, 117, 202, and 243 cm^−1^, in perfect agreement with those obtained by the FLN experiment (39).

After having analyzed the vibrational coherences, we investigate the time scales and pathways of the energy transfer by the global fitting approach (40). To this end, we construct a three-dimensional (3D) dataset by combining a series of 2D electronic spectra at different waiting times. It is found that at least two functions are needed to achieve a converged fitting, and the resulting time constants of the energy transfer are 160 ± 27 fs and 8.8 ± 1.2 ps, respectively. The decay-associated spectra corresponding to these two time constants are depicted in *SI Appendix*, Figs. S6 and S7. It is interesting to note that the time constant of the fastest component of the energy transfer is quite close to the retrieved lifetime of the electronic coherence (see *Discussions*). As a result, one expects that at very low temperature, this fast dynamical component of the energy transfer may have largely been mediated by the electronic quantum coherence. Furthermore, the decay-associated spectra related to the time constant of 8.8 ps (*SI Appendix*, Fig. S7) show clear evidence of a downhill energy transfer from higher-energy excitons to the lowest energy exciton.

### Electronic Quantum Coherence

**Fig. 3.**
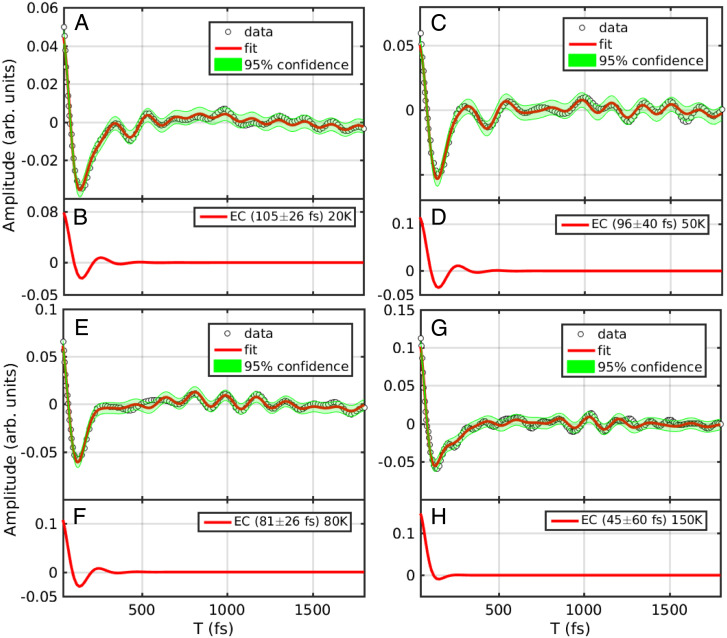
Lifetimes of the measured electronic quantum coherence between excitons 1 and 2 at different temperatures. (*A*) Residuals (black circles) of the cross-peak at ($\omega_{\tau }$, $\omega_{t}$) = (12,120, 12,270) cm^-1^ and the fit by multiexponential functions (red line) at 20K. The green shadow indicates the boundaries of the 95\% confidence interval. (*B*) Retrieved electronic coherence with a decay time constant of 105±26 fs. Residuals and fitted traces at temperatures of 50 K (*C*), 80 K (*E*), and 150 K (*G*). Corresponding electronic coherences with decay time constants of 96±40 fs (*D*), 81±26 fs (*F*), and 45±60 fs (*H*).

To capture the signature of the electronic coherence, we resolve the coherent dynamics of the cross-peak between the two lowest energy excitons, 1 and 2. We choose the cross-peak at (*ω*_*τ*_, *ω*_*t*_) = (12,120, 12,270) cm^−1^ (marked as “CP21” in Fig. 1*B*) to minimize contributions from the energy transfer dynamics. Following the same procedures described in the above section, we extract the residual by first removing the kinetics and then polishing by a Tukey window Fourier transform. The results are shown as black circles in Fig. 3*A*. The oscillations are induced by the mixing of the electronic and vibrational coherence. To distinguish electronic and vibrational coherence, we fit the residual by exponentially decaying sine functions in order to extract the oscillation frequencies and the lifetimes of the coherences. We start the fit by using four frequencies 68, 150, 180, and 202 cm^−1^ obtained experimentally (see *Discussion*), which also agree with the known modes from the FLN experiment (39). In addition, we have obtained an electronic energy gap of 150 cm^−1^ between excitons 1 and 2 by theoretical calculations (see below), in good agreement with previous results (41). Furthermore, one additional frequency of 17 cm^−1^ is included to achieve the best fit with R-square > 0.97, which represents the lowest frequency resolvable within the time steps used. All the fitting procedures are performed using the Curve Fitting Toolbox in Matlab 2013(b), the details of which are given in *SI Appendix*. We show the high-quality fitting results by the red solid line in Fig. 3*A*. The green shadow indicates the boundaries of the 95% confidence interval. This essentially allows us to separate the electronic coherence of 150 cm^−1^ from vibrational coherences. The oscillation originating from the electronic coherence is displayed in Fig. 3*B*, which yields a decay time constant of 105 ± 26 fs. One can readily observe that the electronic coherence sustains over only two oscillation periods and disappears completely within 500 fs. We note that the identified oscillations of the electronic coherence are quite pronounced, larger than 5% of the maximum strength of the 2D spectra at 20 K. We also analyze the coherent dynamics of the cross-peak at (*ω*_*τ*_, *ω*_*t*_) = (12,120, 12,270) cm^−1^ (CP21) for temperatures of 50, 80, and 150 K, and the corresponding traces are plotted in Fig. 3*C*, *E*, and *G*, respectively. The extracted electronic coherences are displayed in Fig. 3*D*, *F*, and *H*, respectively. At 50 K, the electronic coherence lasts less than 500 fs, with a decay time constant of 96 ± 40 fs. Increasing the temperature to 80 K, the lifetime of the electronic coherence is significantly reduced, and we obtain a decay time constant of 81 ± 26 fs. Upon further increasing the temperature to 150 K (see Fig. 3*H*), one can clearly see that the electronic coherence is strongly damped and the oscillation does not survive even a single oscillation period. The decay time constants of electronic coherences at different temperatures are plotted in Fig. 1*J* (marked as “decoherence”). We also retrieve the vibrational coherences at different temperatures by the fitting procedures and show the results in *SI Appendix*.

**Fig. 4.**
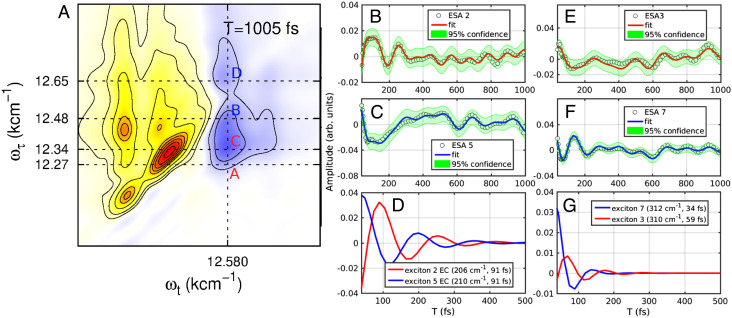
(*A*) Measured 2D electronic spectrum at *T* = 1,005 fs. The ESA peaks of excitons 2, 3, 5, and 7 are labeled as *A*, *C*, *B*, and *D*, respectively. (*B*) Residuals of the ESA peak (black circles) of exciton 2 (red marker A, $\omega_{\tau }=12,270$ cm^-1^) after removing the kinetics by exponential fits. The residuals are further analyzed by fitting functions (red line). (*C*) Residuals (black circles) and fitted traces (blue line) for exciton 5 (blue marker B in (*A*), $\omega_{\tau }=12,480$ cm^-1^). (*D*) The retrieved electronic coherence between excitons 2 and 5 with frequencies of 206±88 and 210±90 cm^-1^. The decay constants are 91±28 fs. The frequencies agree perfectly with the energy gap between excitons 2 and 5. Panels (*E*) and (*F*) show the residuals and fitting traces of excitons 3 and 7. Panel (*G*) gives the retrieved electronic coherences of excitons 3 and 7 with decay time constants of 34±13 and 59±32 fs, respectively. The resolved oscillations show the frequencies of 312±82 and 310±90 cm^-1^, which match the energy difference between excitons 3 and 7.

To study the electronic coherence of exciton states with higher energies, we monitor the dynamics of ESA peaks at *ω*_*t*_ = 12,580 cm^−1^. The energy levels of the excitons 2, 3, 5, and 7 retrieved from our theoretical calculations (see details in the next section) are highlighted by the corresponding *ω*_*τ*_-lines in Fig. 4*A*. The intersection points of two marker lines are denoted as A, B, C, and D, respectively, to which the excited-state dynamics of the excitons 2, 3, 5, and 7 refer. We extract the time evolution of the amplitude of the cross-peaks at A, B, C, and D and remove the underlying kinetics by the global fitting approach. The residuals of the time evolution of those peaks obtained by the Tukey window Fourier transform are depicted as black circles in Fig. 4*B*, *C*, *E*, and *F*, respectively. These residuals are further fitted by exponentially decaying sine functions, and the corresponding high-quality fitting results for excitons 2, 3, 5, and 7 are shown as the red and blue solid lines in Fig. 4*B*, *E*, *C*, and *F*, respectively. More details of the fitting procedure are presented in *SI Appendix*. Let us now focus on the electronic coherence originating from excitons 2 and 5, shown as red and blue solid lines in Fig. 4*D*. We find oscillation frequencies of 206 ± 88 cm^−1^ (exciton 2) and 210 ± 90 cm^−1^ (exciton 5), which are in good agreement with the energy gap between excitons 2 and 5 obtained from our theoretical calculations. Our theoretical calculations further show that the coherence between excitons 2 and 5 is dominated by the strong electronic couplings of pigment 4 and (5,6). of the transformation from the site to the exciton basis are given in *SI Appendix*. Furthermore, it is interesting to note that the well-resolved electronic coherences in Fig. 4*D* exhibit anticorrelated oscillations with a slight phase offset. The electronic coherences originating from excitons 3 and 7 are shown as red and blue solid lines in Fig. 4*G*. We observe an oscillation frequency of ∼310 ± 90 cm^−1^ for both excitons 3 and 7, which agrees well with the energy gap between excitons 3 and 7. Our theoretical analysis shows that this coherence is dominated by the electronic coupling between pigments 1 and 2. As compared to the lifetime of the electronic coherence between excitons 2 and 5, these electronic coherences exhibit smaller time constants of 34 ± 13 and 59 ± 32 fs, which can be attributed to the downhill energy transfer. The larger energy gap between excitons 3 and 5 results in a shorter lifetime of electronic coherence due to the faster energy transfer.

### Theoretical Calculations

We construct a Frenkel exciton model to study the coherent dynamics of the FMO complex. The electronic transitions in the pigments are approximated by transitions between two energy eigenstates, and the electronic couplings between pigments are calculated within the dipole approximation. To account for the fluctuations of the surrounding protein environment, each pigment is linearly coupled to its own thermal reservoir. We consider a spectral density with an overdamped Drude mode and an underdamped mode with a frequency of 180-cm^−1^ to investigate the role of vibrational/vibronic coherences. In passing, we note that the choice of the spectral density is not unique. Yet, the reorganization energy as the integral over all frequencies of the spectral density can serve as a direct and uniquely defined quantifier for the system–bath coupling. The 2D electronic spectra are calculated by a time nonlocal quantum master equation (42–45), and the time evolution of the peaks is obtained by the equation-of-motion phase-matching approach (46). More details are given in the *Materials and Methods* and *SI Appendix*. We use the site energies of the pigments initially from previous works (41) and then optimize them by simultaneously fitting them to the measured absorption spectra at different temperatures. After that, we calculate the 2D electronic spectra and refine the system–bath coupling strengths by comparing the calculated electronic dephasing lifetimes to measured ones at different temperatures. We can thus obtain the optimal set of parameters for the system–bath model by the above procedures.

**Fig. 5.**
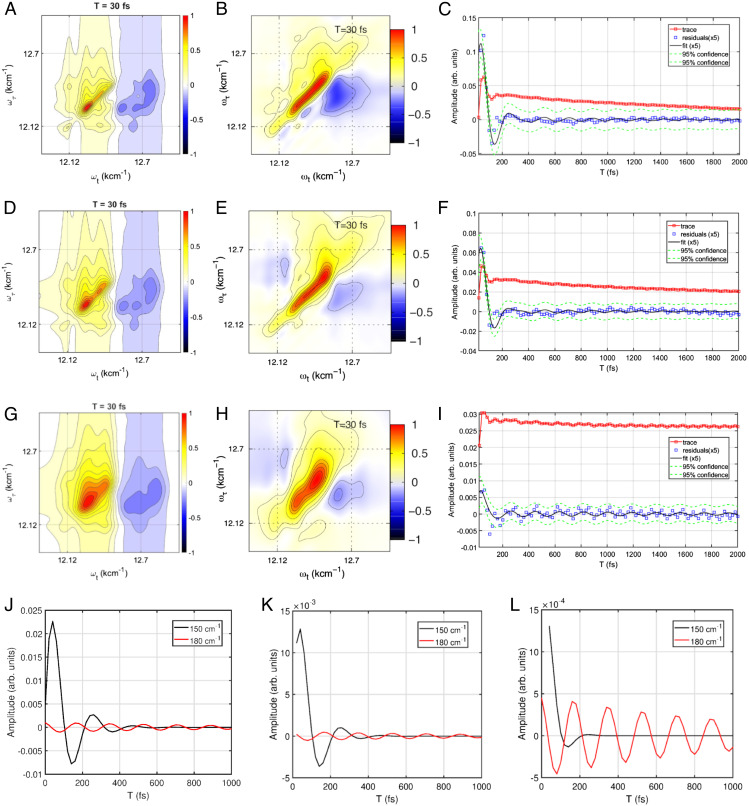
Simulated 2D electronic spectra at 30 fs for temperatures of 50 K (*A*), 80 K (*D*), and 150 K (*G*). Measured spectra at 50 K (*B*), 80 K (*E*), and 150 K (*H*). Time evolutions (red line) of the cross-peak between excitons 1 and 2 (CP21) at temperatures of 50 K (*C*), 80 K (*F*), and 150 K (*I*). The residuals (5 times magnified) are plotted as blue square dots obtained by removing the kinetics. The curve fitting procedure is employed to resolve the oscillations in the residuals, and the fitted data are shown as black solid lines (magnified by 5 times). The fitting quality was quantified by the 95% confidence interval and highlighted by green dashed lines. The retrieved electronic and vibrational coherences are shown as black and red solid lines in (*J*), (*K*), and (*L*), respectively. The electronic coherences decay with lifetimes of 90±22, 80±25, and 45±40 fs at 50, 80, and 150 K, respectively.

We present the simulated 2D electronic spectra at temperatures of 50, 80, and 150 K in Fig. 5*A*, *D*, and *G*, respectively, and the experimental counterparts in Fig. 5*B*, *E*, and *H*, respectively. Despite the complexity in modeling the spectra, the overall agreement between theoretical and experimental results is good. The major discrepancy between theory and experiment arises from the higher-energy excitonic states, which are mainly caused by the delta-pulse approximation employed in the simulation (*SI Appendix*, section XIV). The laser profile used in our measurement, on the other hand, covers mainly the high-frequency part of the absorption spectrum, with a central frequency at 13,200 cm^−1^ (*SI Appendix*, Fig. S1). We show the simulated time evolution of the cross-peak at (*ω*_*τ*_, *ω*_*t*_) = (12,120, 12,270) cm^−1^ (marked as CP21 in Fig. 1*B*) for temperatures of 50, 80, and 150 K as red lines in Fig. 5*C*, *F*, and *I*, respectively. We then analyze the calculated data by the global fitting approach as described above. The retrieved residuals (magnified 5 times) are plotted as blue square dots in Fig. 5*C*, *F*, and *I*. We employ the fitting procedure to analyze the coherent dynamics of those residuals. To this end, we choose the frequencies of 150 and 180 cm^−1^ to characterize the electronic quantum coherence between excitons 1 and 2 and the vibrational coherence, respectively. The fitting procedures are performed using the Curve Fitting Toolbox in Matlab, the details of which are illustrated in the *SI Appendix*, section IX. We present the fitted traces as black solid lines with the 95% of confidence interval (green dashed lines) in Fig. 5*C*, *F*, and *I*. The resolved electronic and vibrational coherences are shown as red and black lines, respectively, in Fig. 5*J*–*L*, respectively. At 50 K (Fig. 5*J*), it is found that while the electronic coherence lasts for two oscillation periods and has disappeared at 500 fs, one can clearly see the long-lived vibrational coherence. We further note that the oscillation phase of the electronic coherence retrieved from Fig. 5*J* matches quite well with that revealed by the experimental data in Fig. 3*D*. By analyzing the electronic and vibrational coherences at different temperatures, we conclude that while the lifetimes of the electronic coherence are dramatically reduced with the increase of the temperature, the vibrational coherences are still rather robust when increasing temperature, consistent with experimental results. The same conclusion can be drawn for the higher-energy excitons at different temperatures, the details of which are shown in *SI Appendix*.

## Discussion

An important question related to the exciton transfer dynamics is the nature of the bath-induced fluctuations. It can be characterized by the measure of the non-Markovianity which quantifies how strongly the dephasing and relaxation dynamics deviate from the Markovian, i.e., memory-less behavior. In general, a highly structured environment consisting of several strongly coupled modes of the FMO protein may give rise to significant non-Markovian dynamics. Early numerically exact path-integral calculations (47) on the basis of an experimentally determined spectral density have shown that the exciton dynamics is purely Markovian at ambient conditions. This finding has been recently confirmed experimentally by comparing the decay time of the optical dephasing and electronic quantum coherence in the FMO complex (24). The equivalence of the time scales of the optical dephasing and the electronic decoherence reveals that the dynamics of the energy transfer in the FMO complex at room temperature is fully Markovian.

Here, we provide a complete picture of the role of the non-Markovianity in the FMO complex at different temperatures. As discussed above, the extracted lifetime of the electronic between two lowest-energy excitons at 20 K exhibits a decay time of 105 ± 26 fs, whereas the analysis of the antidiagonal bandwidth yields a decay time of 197 ± 8 fs for the optical dephasing of exciton 1. The difference of almost a factor of 2 is due to the lower temperature but is still covered by a fully Markovian description of the transfer dynamics (48). This finding is also in agreement with low-temperature calculations (47). On the basis of these studies, we conclude that non-Markovian energy fluctuations of the pigments induced by pigment-hosted molecular vibrations do not play a role in the energy transfer of the FMO complex. Despite its simple structure, we believe that this conclusion can be extended to more complicated photosynthetic protein complexes.

Instead of long-lived electronic coherence, our study uncovers long-lasting beating dynamics induced by vibrational coherences with frequencies around 180 cm^−1^ in the electronic ground state. As summarized in the Introduction section, several studies have suggested a resonant enhancement of the short-lived electronic coherence by the long-lived vibrational coherence in the FMO complex. However, our work clearly illustrates that there is no such resonant enhancement of the electronic coherence during the population transfer. In contrast, we have retrieved a reorganization energy of 120 cm^−1^ from the measured and calculated 2D electronic spectra, which manifests a quite strong system–bath interaction that can rapidly destroy the phase of the electronic quantum coherences between pigments in the FMO complex. We have also explored the coherent dynamics with a smaller reorganization energy of 35 cm^−1^, and we obtained a longer lifetime of 137 fs for the electronic quantum coherence, which is inconsistent with the measured results at 50 K. The details are presented in *SI Appendix*, section XVI.

A final comment regarding the magnitude of the reorganization energy is in order. The (too small) value of 35 cm^−1^ emerges when only the intramolecular vibrational protein modes are selected to contribute and the electronic background continuum is neglected. In fact, starting from the spectral density of Adolphs and Renger (49), which is based on the FLN spectra of Wendling et al. (50) and which has been parametrized in a particular super-Ohmic form plus a 180-cm^−1^ distinct vibrational mode, the energy can be trivially calculated as the integral over the entire frequency range. One finds that the continuous electronic background contributes a magnitude of *E*_*R*,el_  =  60 cm^−1^, while the 180-cm^−1^ mode contributes with *E*_*R*,vib_  =  39.6 cm^−1^. Note that Kell et al. (51) argued that the latter value is slightly too large because the Huang–Rhys factor would be smaller. This led to the use of the 35 cm^−1^ as was the consensus in the field. Both contributions combine to the sum of 99.6 cm^−1^, which is much closer to *E*_*R*_=120 cm^−1^ retrieved from our measured data.

Hence, instead of the energy transfer being enhanced by strongly delocalized exciton wave functions, a rather large reorganization energy sharpens the limits for the delocalization and the energy pathways between pigments. As a result, the efficient downhill transfer of partially delocalized excitons is determined by a simple thermal distribution of excitons, which rapidly arise from the initial ultrafast nonequilibrium dynamics triggered by the photoexcitation.

## Conclusions

In this paper, we provide a complete picture of the coherent contribution to the energy transfer in the FMO complex by 2D electronic spectroscopy in the entire regime from low to high temperatures. In particular, the spectroscopic measurements at a low temperature of 20 K allow us to provide unambiguous evidence of the lifetime of the electronic quantum coherence and to disentangle the electronic coherence from long-lived vibrational coherence. Interestingly, due to the downhill energy transfer, the electronic coherence between the two lowest excitons marginally persists out to 500 fs at 20 K. However, the coherence lifetime of higher excitons is dramatically reduced by the population transfers. This analysis allows us to disentangle the previously reported long-lived beating of cross-peak signals and to show that they are composed of mixed ground-state molecular Raman modes. Moreover, we uncover that the lifetime of electronic coherence is significantly modulated by temperature, while, in contrast, the resonant beatings of vibrational coherences last for picoseconds even at 150 K. A thorough analysis on the basis of a unique combination of experimental data and theoretical modeling enables us to provide a reliable estimate for the decisive parameter of the reorganization energy of the FMO complex. We find a reorganization energy of 120 cm^−1^, which represents a strong system–bath interaction of the pigments with their protein environment. This coupling is sufficient to significantly reduce the lifetime of electronic coherences and leads to a rapid intermittent localization of the electronic wave function on a few molecular sites. Instead of a long-lived quantum coherent energy transfer, we provide a different picture of a downhill energy transfer, in which the pathways of population transfers are dynamically constructed simply by following lower site energies of the pigments involved and by rather fast electronic damping due to significant nuclear reorganization in the excited state. The latter sharpens the funnel directing the energy flow in the FMO complex. In general, we conclude that the energy transfer in the FMO complex is dominated by the thermal dynamics of weakly delocalized excitons after an initial ultrafast nonequilibrium photon excitation. Due to common features in the bath for all light-harvesting systems, despite the relative simplicity of FMO, we believe that this conclusion can be further extended to the other more complicated photosynthetic protein complexes.

### Sample Preparation

The FMO protein was isolated from the green sulfur bacteria *Chclorobaculum tepidum* (see *SI Appendix* for more details). The sample was dissolved in a Tris buffer at pH 8.0. It was filtered with a 0.2-μm filter to reduce light scattering. The sample was then mixed 70:30 v/v in glycerol and kept in a homebuilt cell with an optical pass length of 500 μm. The cell was mounted in the cryostat (MicrostatHe-R) for the low-temperature measurements.

### 2D Electronic Measurements with Experimental Conditions

Details of the experimental setup have already been described in earlier reports from our group (24). Briefly, the measurements have been performed on a diffractive optics-based, all-reflective 2D spectrometer, with a phase stability of *λ*/160. The laser beam from a homebuilt nonlinear optical parametric amplifier (NOPA; pumped by a commercial femtosecond Pharos laser from Light Conversion) is compressed to ∼16 fs using the combination of a deformable mirror (OKO Technologies) and a prism pair. Frequency-resolved optical grating (FROG) measurement is used to characterize the temporal profile of the compressed beam, and the obtained FROG traces are evaluated using a commercial program FROG3 (Femtosecond Technologies). A broadband spectrum so obtained carried a linewidth of ∼100 nm full width at half maximum (FWHM) centered at 750 nm. Three pulses are focused on the sample with the spot size of ∼100 μm, and the photon echo signal is generated in the phase-matching direction. The photon echo signals are collected using a Sciencetech spectrometer model 9055 which is coupled to a CCD linear array camera (Entwicklungsbüro Stresing). The 2D spectra for each waiting time *T* were collected by scanning the delay time *τ* = *t*_1_ − *t*_2_ in the range of [−200 fs, 350 fs] with a delay time step of 2 fs. At each delay step, 100 spectra were averaged to reduce the noise. The waiting time *T* = *t*_3_ − *t*_2_ was linearly scanned in the range of 2.0 ps in steps of 15 fs. For all measurements, the energy of the excitation pulse is attenuated to 8 nJ with 1 kHz repetition rates. Phasing of the obtained 2D spectra was performed using an “invariance theorem” (52).

### Theoretical Calculations

A Frenkel exciton model is constructed to calculate the coherent energy transfer and the 2D electronic spectra of the FMO complex. The total Hamiltonian is constructed in the form of the system, bath, and system–bath interaction terms, *H* = *H*_*S*_ + *H*_*B*_ + *H*_*SB*_. The system Hamiltonian is given as $H_{S}=\epsilon_{g}|g\rangle \left\langle g|+\sum_{m}^{N}\epsilon_{m}|m\right\rangle \left\langle m|+\sum_{l,m\neq l}^{N}J_{\mathrm{ml}}|m\right\rangle \langle l|$, where *ϵ*_*g*_ and *ϵ*_*m*_ are the site energies of ground and *m*th excited pigment, respectively. *J*_*ml*_ is the electronic interaction between the *m*th and *l*th pigments. *N* = 7 pigments are included in the monomeric FMO complex. Moreover, each pigment is coupled to its own individual bath. The bath Hamiltonian can be written as $H_{B}=\sum_{m}^{N}\sum_{\xi }\hbar \omega_{m\xi }b_{m\xi }^{†}b_{m\xi }$, where $b_{m\xi }^{†}\left(b_{m\xi }\right)$ is the creation (annihilation) operator of the *ξ*th fluctuation mode associated with site *m* and its angular frequency *ω*_*mξ*_. *H*_*SB*_ = ∑_*m*_*V*_*m*_*W*_*m*_ describes the interaction of the system with the bath, where we have defined *V*_*m*_ = |*m*⟩⟨*m*| and $W_{m}=-\sum_{\xi }c_{m\xi }\left(b_{m\xi }^{†}+b_{m\xi }\right)$. The *c*_*mξ*_ is the coupling constant between the *m*th pigment and *ξ*th fluctuation mode. The bath is specified by the spectral density *J*_*m*_(*ω*)=*π*∑_*ξ*_ℏ^2^*c*_*mξ*_^2^*δ*(*ω* − *ω*_*mξ*_). We include one overdamped mode and one underdamped mode to study the impact of vibrational coherence. The corresponding spectral density can be expressed as


$$\begin{array}{c} J\left(\omega \right)=\frac{2\Lambda \Gamma \omega }{\omega^{2}+\Gamma^{2}}+\frac{4S\gamma_{\mathrm{vib}}\omega_{\mathrm{vib}}^{3}\omega }{{\left(\omega^{2}-\omega_{\mathrm{vib}}^{2}\right)}^{2}+4\gamma_{\mathrm{vib}}^{2}\omega^{2}}. \end{array}$$

Here, *Λ* and *Γ*^−1^ are the damping strength and the bath relaxation time of the overdamped mode, respectively. *S*, *ω*_vib_, and $\gamma_{\mathrm{vib}}^{-1}$ are the Huang–Rhys factor, the vibrational frequency, and the vibrational relaxation time of the underdamped mode, respectively. This form has been shown to describe the experimental data (24) correctly.

The nonequilibrium dynamics of the system–bath model is calculated by a time-nonlocal quantum master equation, the details of which are described in *SI Appendix*. The linear response theory is used to calculate the absorption spectrum of the FMO complex, *I*(*ω*)=⟨∫_0_^∞^*dte*^*iωt*^tr(**μ**(*t*)**μ**(0)*ρ*_*g*_)⟩_rot_, where *ρ*_*g*_ = |*g*⟩⟨*g*| and a *δ*-shaped laser pulse is assumed. ⟨ ⋅ ⟩_rot_ denotes the rotational average of the molecules with respect to the laser direction. Moreover, the 2D electronic spectra are obtained by calculating the third-order response function


$$\begin{array}{cc} S^{\left(3\right)}\left(t,T,\tau \right) & ={\left(\frac{i}{\hbar }\right)}^{3}\Theta \left(t\right)\Theta \left(T\right)\Theta \left(\tau \right)\\ & \;\times \mathrm{tr}\;\left(\mu \left(t+T+\tau \right)\;\left[\mu \left(T+\tau \right),\left[\mu \left(\tau \right),\left[\mu \left(0\right),\rho_{g}\right]\right]\right]\right). \end{array}$$

Here, *τ* is the delay time between the second and the first pulse, *T* (the so-called waiting time) is the delay time between the third and the second pulse, and *t* is the detection time. To evaluate 2D electronic spectra, we need the rephasing (RP) and nonrephasing (NR) contributions of the third-order response function, i.e., $S^{\left(3\right)}\left(t,T,\tau \right)=S_{\mathrm{RP}}^{\left(3\right)}\left(t,T,\tau \right)+S_{\mathrm{NR}}^{\left(3\right)}\left(t,T,\tau \right)$. Assuming the impulsive limit (the *δ*-shaped laser pulse), one obtains


$$\begin{array}{cc} I_{\mathrm{RP}}\left(\omega_{t},T,\omega_{\tau }\right) & =\int_{-\infty }^{\infty }d\tau \int_{-\infty }^{\infty }dt\;\;e^{i\omega_{t}t-i\omega_{\tau }\tau }S_{\mathrm{RP}}^{\left(3\right)}\left(t,T,\tau \right),\\ I_{\mathrm{NR}}\left(\omega_{t},T,\omega_{\tau }\right) & =\int_{-\infty }^{\infty }d\tau \int_{-\infty }^{\infty }dt\;\;e^{i\omega_{t}t+i\omega_{\tau }\tau }S_{\mathrm{NR}}^{\left(3\right)}\left(t,T,\tau \right). \end{array}$$

The total 2D signal is the sum of the two, i.e., *I*(*ω*_*t*_, *T*, *ω*_*τ*_)=*I*_RP_(*ω*_*t*_, *T*, *ω*_*τ*_)+*I*_NR_(*ω*_*t*_, *T*, *ω*_*τ*_).

The model parameters of the site energies and electronic couplings are initially taken from ref. (41), and the site energies are further refined during a simultaneous fit to the absorption spectra of the FMO complex at different temperatures. To precisely determine the reorganization energy, the parameters are further refined by fitting to the experimental antidiagonal bandwidth of the main peak of exciton 1.

## Supplementary Material

Appendix 01 (PDF)

## Data Availability

All study data are included in the article and/or *SI Appendix*.
