# Supplementary material for: Quantum coherent energy transport in the Fenna–Matthews–Olson complex at low temperature
Source: Proc Natl Acad Sci U S A. 2022 Nov 28;119(49):e2212630119. doi: 10.1073/pnas.2212630119 (PMC9894199; doi:10.1073/pnas.2212630119)
Supplement: Supplementary file 1 — Appendix 01 (PDF) [file pnas.2212630119.sapp.pdf]

Supporting Information for

Quantum Coherent Energy Transport in the Fenna-Matthews-Olson Complex at Low Temperature

H.-G. Duan, A. Jha, L. Chen, V. Tiwari, R. J. Cogdell, K. Ashraf, V. I. Prokhorenko, M. Thorwart and R. J. D. Miller

Corresponding Authors: Hong-Guang Duan, Michael Thorwart, R. J. Dwayne Miller.
E-mail: duanhongguang@nbu.edu.cn, michael.thorwart@physik.uni-hamburg.de, dmiller@lphys.chem.utoronto.ca

This PDF file includes:

Supporting text
Figs. S1 to S39
Table S1
SI References

Supporting Information Text

In this document, we first show the measured two-dimensional (2D) electronic spectra of the Fenna-Matthews-Olson (FMO) complex at different temperatures. Then, we describe the details of the procedure to retrieve the anti-diagonal bandwidth. The Tukey window Fourier transform and the wavelet analysis are described in Secs. V and VI. We present the global fitting approach and analyze the decay-associated spectrum (DAS) and the 2D power spectra of the FMO complex in Sec. VII. After this, we describe the process to identify the electronic coherence from vibrational signals. We show the details of the results obtained from the curve fitting toolbox. The identified electronic coherence between excitons of higher energies are shown in Sec. X. In addition, the basis transformation from the localized pigment to the exciton basis is presented in Sec. XII. Finally, the theoretical simulations of the 2D spectra and the quantum master equation are described in Secs. XIV and XIX.

I. Preparation of the FMO protein

The Fenna-Matthews-Olson (FMO) protein complex was prepared from cells of the thermophilic green sulfur bacterium *Chlorobium tepidum*. A strain of *C. tepidum* that had been engineered to produce a His-tagged version of its reaction center was used in order to simplify the purification strategy. This strain was a gift of Prof. Oh-Oka (1). The cells were grown anaerobically in the light in modified Pfennig's media (2). New cultures were allowed to go fully anaerobic in the dark overnight, then they were grown at a light intensity of about 30 micromoles photonsmm^{-2}s^{-1} at 43°C for 2 to 3 days. When fully grown, the cells were harvested by centrifugationat 12,000 x g for 20 min. The reaction centres (which bind FMO) were isolated from brokencells (3). At the stage of the nickel affinity column chromatography a high salt wash at 500 mM NaCl elutes the FMO complexes. This dilute solution of FMO was then purified by a combination of ion exchange chromatography on a Whatrman DE52 cellulose resin and size exclusion chromatography on a Sepharose S-200 column.

II. Absorption spectrum and measuring laser

In this section, we show the measured absorption spectrum at a temperature of 80 K in Fig. S1. Moreover, the measuring laser spectrum is depicted as light blue shadow.

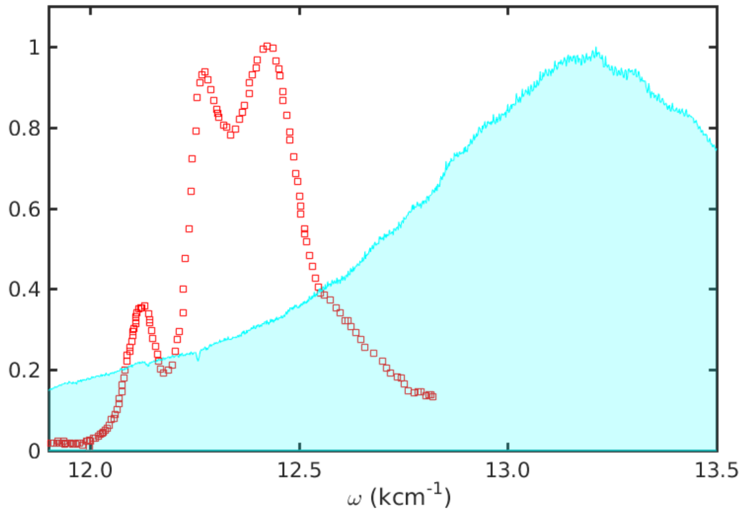

Fig. S1. Absorption spectrum of the FMO complex at 80 K. The measuring laser is shown as light blue shadow.

III. Two-dimensional electronic spectra at 50, 80 and 150 K

In this section, we show the measured 2D electronic spectra of the FMO complex for different temperature. In Fig. S2, the real parts of the 2D electronic spectra were measured at 50 K. The selected waiting times are 30, 50, 510 and 1005 fs, respectively. The positive and negative magnitudes in the 2D spectra indicate the transitions associated to the ground-state-bleach (GSB) and the excited-state-absorption (ESA), respectively. The measured spectra at 80 and 150 K are shown in Fig. S3 and Fig. S4, respectively.

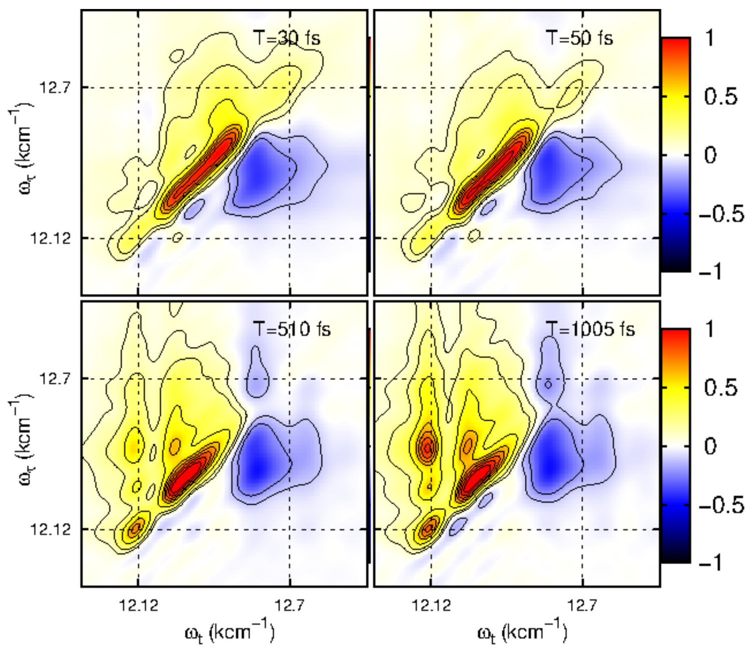

Fig. S2. Real parts of the 2D electronic spectra of the FMO complex measured at 50 K. The selected waiting times are 30, 50, 510 and 1005 fs, respectively.

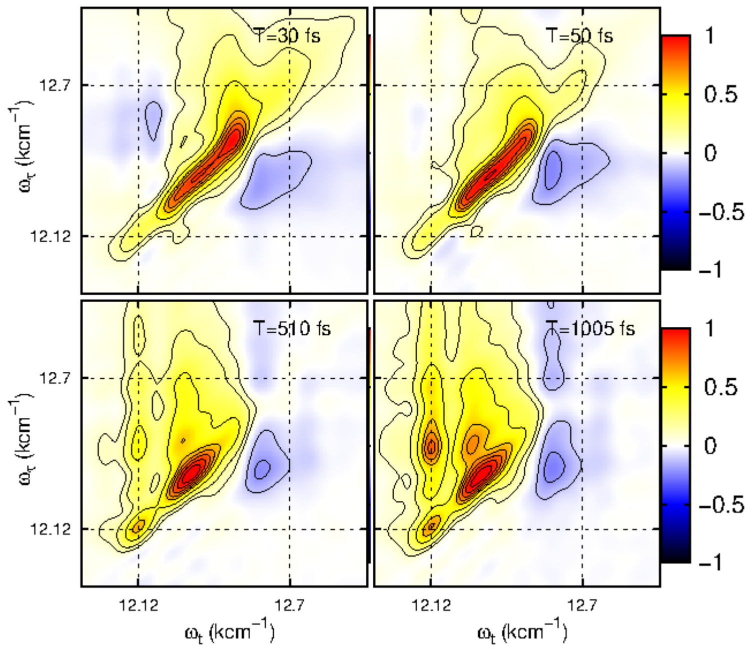

Fig. S3. Real parts of the 2D electronic spectra of the FMO complex at 80 K.

IV. Fitting procedure of the anti-diagonal bandwidth

In this section, we introduce the procedure to extract the timescales of electronic dephasing. It is known that the decay time constant associated to electronic dephasing can be directly extracted from the anti-diagonal bandwidth in 2D spectra for zero initial population time. Here, we extract the decay time constant by measuring the anti-diagonal bandwidth of the lowest-energy main peak ($\omega_\tau = \omega_t = 12120$ cm^{-1}) in the 2D spectra. Based on the theory of the lineshape function (4), the dominant contribution to the anti-diagonal bandwidth stems from the damping of the oscillations due to the energy gap between the ground and excited states after photo-excitation. We estimate the bandwidth of the peak by fitting the signal to a Lorentzian lineshape.

We analyze the 2D spectrum of the waiting time at 30 fs to remove the pulse overlap effect at initial time. We first extract

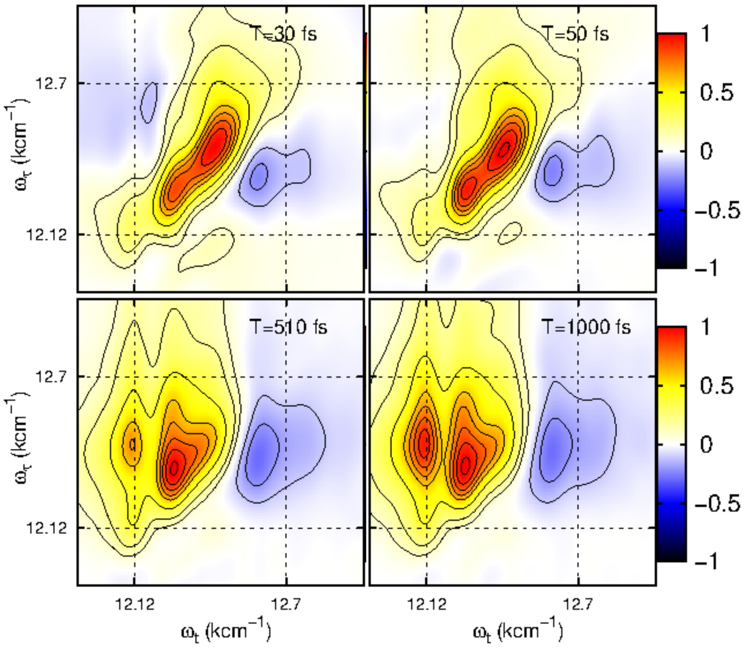

Fig. S4. Real parts of the 2D electronic spectra of the FMO complex at 150 K.

the anti-diagonal profile of the main peak (exciton 1) along the black line marked in Fig. S5(a). The retrieved profile is shown in Fig. S5(b) as black solid line. Three Lorentzian lineshape functions are used to obtain the converged fit, which is plotted as red dashed line. The initial values of the fitted peaks are centered at 12000, 12120 and 12180 cm^{-1}, respectively. The fitting process is finished using the curve fitting toolbox in Matlab 2013(b). The time constant of the electronic dephasing can be obtained from the fitted bandwidth, $\tau_{\text{hom}} = (\pi c \Delta_{\text{hom}})^{-1}$. By this, we obtain the decay time constants of the electronic dephasing as $\sim$ 197±8 fs at 20 K. We repeat the same procedure for the profiles at 50, 80 and 150 K and obtain the decay time constants of the electronic dephasing as 181±9, 147±7 and 75±10 fs, respectively. The anti-diagonal profiles at 50, 80 and 150 K and the resulting fitting curves are shown in Fig. 1 of the main text.

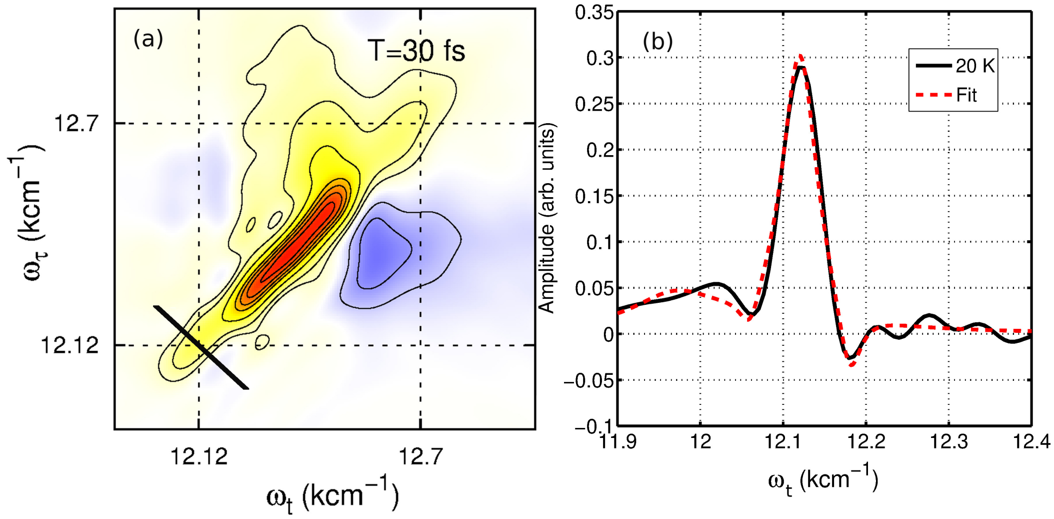

Fig. S5. (a) Real part of the 2D electronic spectrum of the FMO complex at a waiting time of 30 fs measured at 20 K. The anti-diagonal bandwidth is extracted from the main peak of exciton 1, which is marked as a black line. The projection along ω_t is plotted as black solid line in (b). Three Lorentzian lineshapes are used to obtain a high-quality fit and the obtained trace is shown as a red dashed line in (b). The anti-diagonal bandwidth of the main peak at $\omega_\tau = \omega_t = 12120$ cm^{-1} yields a decay time constant of 197±8 fs at 20 K.

V. Tukey window Fourier transform

Here, we provide the details of the Fourier transform with the Tukey window. To isolate the high-frequency jitters, Fourier filtering in the frequency domain is employed. By this, we separate each of these regions of interest with the Tukey window, which has the form

$$\omega(n) = \begin{cases} 1, & 0 \le |n| \le \alpha \frac{N}{2} \\ \frac{1}{2}\left(1 + \cos\left[\frac{\pi(n-\alpha\frac{N}{2})}{(1-\alpha)\frac{N}{2}}\right]\right), & \alpha\frac{N}{2} \le |n| \le \frac{N}{2} \end{cases} . \quad \text{[S1]}$$

Due to the flat top, it conserves the amplitudes of the Fourier components of interest over a frequency range larger than a cosine or a Gaussian window, while it still limits the artifacts arising from a pure bandpass filter. In this work, we use the Tukey window with $\alpha = 1/5$ and a Fourier bandpass filter with $<1000\ \mathrm{cm}^{-1}$.

VI. Wavelet analysis

In this section, we summarize the basic principles of the wavelet transform, the details of which have been described in Refs. (5, 6). It starts from the definition of a zero-mean and short-time oscillating function ψ, called a "mother" wavelet, which is used to decompose a one- or multi-dimensional real-valued signal into different frequency bands. This mother wavelet function is translated in time by t and stretched by the scaling factor ω^{-1}, giving the wavelet "atom" function

$$\psi_{t,\omega}(t') = \sqrt{\omega}\psi\left([t'-t]\omega\right) . \quad \text{[S2]}$$

It provides the effective basis for the transformation. The two most common transforms are the discrete wavelet transform and the continuous wavelet transform CWT (7). The discrete one decomposes the signal into several frequency bands and is frequently used for data and image compression. The continuous one, which is used in this paper, is based on an expansion of a temporal signal $f(t)$ via the inner product of the function with a wavelet atom,

$$CWT_f(t,\omega) = \int_{-\infty}^{+\infty} dt' f(t')\sqrt{\omega}\psi^*\left([t'-t]\omega\right) . \quad \text{[S3]}$$

The parameter t indicates where the wavelet atom is centered, while the scale parameter ω^{-1} controls the relative width of the wavelet atom compared to the mother wavelet function. This nonlinear integral transform provides a high time resolution of high-frequency components, while for the slowly varying components of the signal, the frequency resolution is high. It projects the signal onto basis functions with a varying "center" frequency and a varying range fixed by the scaling factor.

VII. Global fitting approach and decay associated spectra

In this section, we describe the data analysis by the global fitting approach. In this method, a sequence of 2D spectra are collected to form the three-dimensional (3D) array $\mathrm{S}(\omega_\tau, \mathrm{T}, \omega_\mathrm{t})$. This 3D data set is then decomposed into a sum of 2D decay-associated spectra $\mathrm{A}(\omega_\tau, \omega_\mathrm{t})$ with the individual exponential decay with the lifetime τ_i according to

$$S(\omega_\tau, T, \omega_t) = \sum_i A_i(\omega_\tau, \omega_t)\exp(-T/\tau_i). \quad \text{[S4]}$$

To avoid a pulse overlap effect, we perform the fit at the starting time of 30 fs. The measured window extends up to 2 ps with a time step of 15 fs. To achieve a good fit, at minimal two exponential functions are used to fit the kinetics in the 3D data. The obtained decay-associated spectrum is shown in Fig. S6 with the decay time constant of 160 fs. In Fig. S6, two positive and two negative peaks are shown along the diagonal. Moreover, two cross peaks are presented in the upper-left part with negative magnitude. It is well known that the positive (negative) peak in decay-associated spectrum manifests the exponential decay (increase) of the magnitude in 2D electronic spectra with varying waiting time. For clarity, theory-resolved excitonic positions are marked by a dashed grid in Fig. S6. First, we observe that the main peaks fit well to the excitonic positions. This verifies that this component maps the energy transfer between excitons. Second, the decay time constant of this component is 160 fs, which is comparable to the timescale of the electronic quantum coherence in the FMO complex at 20 K. Based on this observation, we believe that the fast energy transfer revealed by this component is fully mediated by electronic coherence. Third, multiple pathways of the energy transfer are resolved in the FMO complex: (a) A large portion of the transfer of population from higher-energy excitonic states (4, 5, 6, 7) to the second exciton state is visible. This energy-transfer pathway is apparent by two cross peaks in the upper-left part (ω_t=12270 cm^{-1}). (b) A small portion of energy transfer from exciton 7 to 3 is present, which is identified by the negative magnitude of the main peak at exciton 3. (c) Finally, energy transfer from exciton 2 to 1 occurs. The positive and negative peaks of exciton 2 and 1 indicate the population transfer between them. Moreover, the increase of the cross peak at (ω_τ, ω_t) = (12270, 12120) cm^{-1} manifests the energy transfer from exciton 2 to 1.

In addition, one more component with a decay time constant of 8.8 ps is needed to achieve a good fit, which is shown in Fig. S7. In this work, we only measure the 2D spectra of the FMO complex up to 2 ps, which apparently is shorter than the energy transfer occuring at longer timescales. Thus, this component of 8.8 ps can be considered as the infinity component in this fit.

3D residuals are obtained after removing the kinetics obtained from the global fitting approach. We perform the Fourier transform of 3D residuals along the waiting time T. The resulting 2D patterns for particular frequencies are shown in Fig. S8.

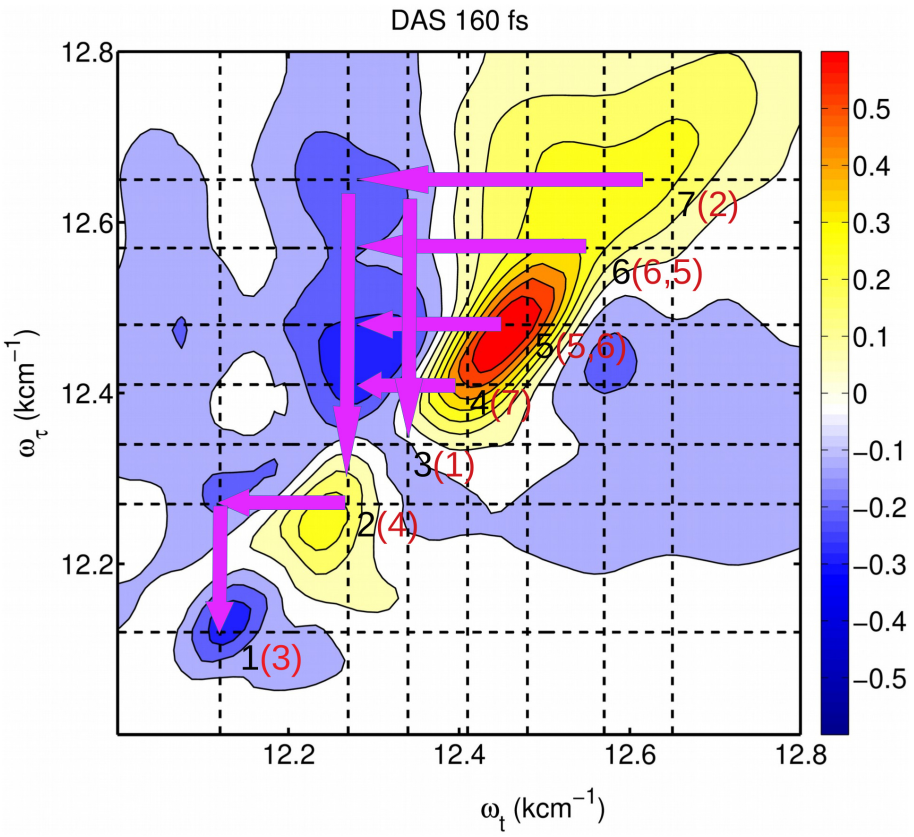

Fig. S6. Decay-associated spectrum of the FMO complex at 20 K for the decay time constant of 160 fs. Theory-resolved exciton spectral positions are marked as dashed grid. The excitonic states (site states) are marked as black (red) numbers.

The power spectra of the modes at 151, 170, 207 and 245 cm^{-1} are shown in (a), (c), (d) and (e), respectively. In (a), this 2D power spectrum shows a strong peak at $(\omega_\tau, \omega_t) = (12120, 12270)$ cm^{-1}, which can be associated with the excitonic states of 1 and 2. Moreover, the oscillation period of this mode perfectly agrees with the energy gap between exciton 1 and 2. By this, we believe that this strong cross peak is the direct evidence of electronic dynamical coherence between the two lowest-energy excitons. The magnified picture is shown in (b) focussing to the range of exciton 1 and 2. Moreover, in (a), two strong cross peaks are visible at $(\omega_\tau, \omega_t) = (12380, 12250)$ and $(12500, 12270)$ cm^{-1}, respectively. To understand these two cross peaks, we need to examine the power spectrum of the neighboring frequency at 170 cm^{-1}. In (c), the 2D power spectrum at 170 cm^{-1} shows even stronger cross peaks in the same range at $(\omega_\tau, \omega_t) = (12380, 12250)$ and $(12500, 12270)$ cm^{-1}. More interesting, the 2D power spectrum of 207 cm^{-1} shows strong cross peaks in the same frequency range as well. It is well known that both modes (170, 207 cm^{-1}) are the Raman modes of the electronic ground state of the FMO complex (8). To show the difference in the amplitude, we plot the power spectrum of the resolved low-frequency modes in (f). We find the strongest amplitude of peaks for the frequencies 170 and 202 cm^{-1} (the slight difference of the modes compared to the 2D power spectrum is due to the different time window of the Fourier transform). The mode at 150 cm^{-1} is close to the vibrational mode at 170 cm^{-1} and both strongly overlap with each other, such that it becomes difficult to separate both modes. Based on this finding, we interpret the cross peaks $(\omega_\tau, \omega_t) = (12380, 12250)$ and $(12500, 12270)$ cm^{-1} in (a) as mainly consisting of the contribution of the mode at 170 cm^{-1}. Moreover, the 2D power spectra of the close-by frequency modes at 170 and 207 cm^{-1} show strong peaks at the same position, which induces a resonant beating of vibrational coherence. In addition, the power spectrum of 245 cm^{-1} is plotted in (e), which agrees well with the Raman mode measured in the FLN experiment (8).

VIII. Beatings of vibrational coherences in cross peaks

In this section, we examine the resonant beatings of vibrational coherences. For this, we first extract the magnitude of the cross peaks from the 2D electronic spectra at different waiting times. The positions of the cross peaks are identified using the eigenenergies in our system Hamiltonian. We further obtain the residuals of the oscillations by removing the kinetics employing the global fitting approach. The coherent dynamics of these vibrational coherences is then examined by the wavelet analysis. The obtained results of the vibrational beatings are shown in Figs. S9 to S12.

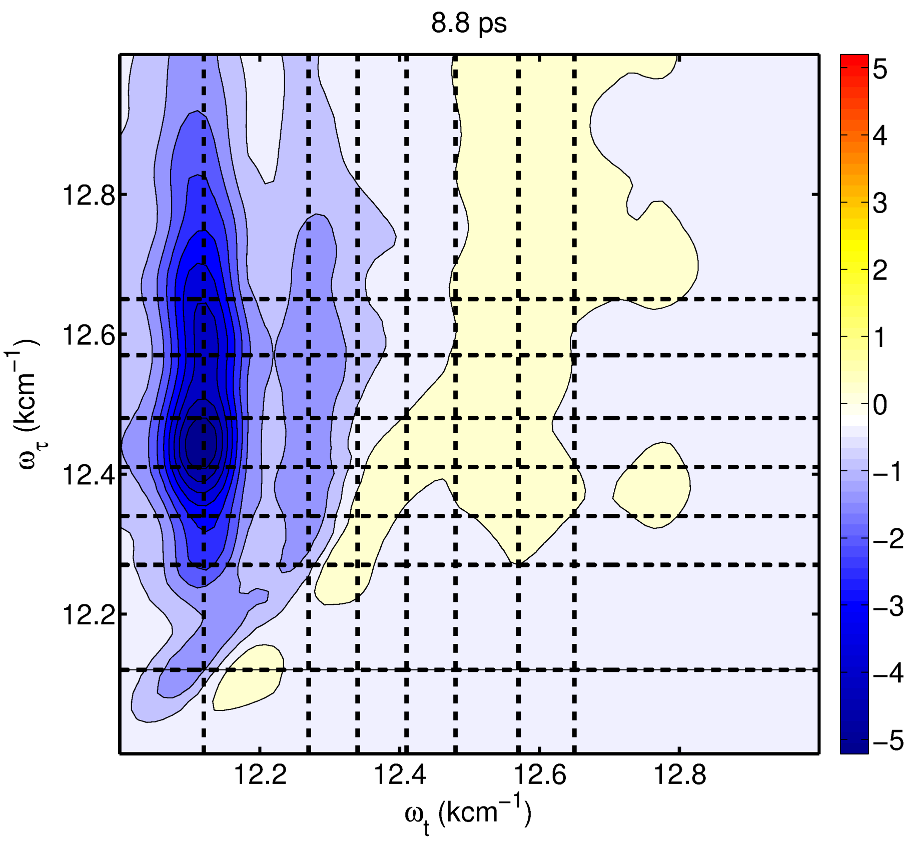

Fig. S7. Same as in Fig. S6, but for the decay time constant of 8.8 ps.

IX. Identifying the electronic coherence between exciton 1 and 2

In the previous section, we have examined the 2D power spectra of the FMO complex. We have explained the strong overlap between oscillations at frequencies of 150 and 170 cm^{-1} due to the strong frequency gap. An effective tool to identify the lifetime of the electronic coherence from the vibrations still needs to be provided. Here, we present a new way to separate the electronic coherence from the vibrations between exciton 1 and 2.

In Fig. S13(a), we show the raw trace (red solid line) of the cross peak signal associated to the transition between exciton 1 and 2 at $(\omega_\tau, \omega_t) = (12120, 12270)$ cm^{-1}. First, the kinetics is removed by a fit to an exponential function, which is plotted as a black dashed line in (a). Subsequently, the obtained residual is plotted as red solid line in (b). To remove high-frequency noise, a Tukey window Fourier transform is performed with the parameters $\alpha = 1/5$ and $<$1000 cm^{-1} (more details are given in Sec. V). The polished oscillations are plotted as black dashed line in Fig. S13(b). After this, this Fourier filtered trace is uploaded into the curve fitting toolbox in Matlab 2013(b). The fitting function is given as

$$\sum_{i}^{N} A_i e^{-T/\tau_i} \sin(\omega_i T + \phi_i). \qquad \text{[S5]}$$

To separate electronic coherence at the frequency of 150 cm^{-1}, five oscillatory components (N=5) are used to fit the trace. They are given by the modes at the frequencies 15, 68, 150, 170 and 201 cm^{-1}, respectively. Among them, 68, 170 and 201 cm^{-1} are the Raman modes resolved as described in the previous section. Moreover, one low-frequency mode of 15 cm^{-1} is used to remove the slow fluctuations of the laser output power. To set the proper initial values, we restrict the mode frequencies in a range around 5 cm^{-1} and fix the initial guess of the lifetimes identically to 500 fs, with a range from 0 to ∞. With these parameters, we obtain a high quality fit with an R-square $\geq$0.98 from the curve fitting toolbox. The trace and the obtained fits are shown as black circles and red solid line in Fig. S14 with a confidence range of 95%. Here, to achieve a good fit, the first time point has been excluded in the fitting procedure. By this, we separate the electronic coherence from ground-state vibrations. The frequency components and its associated decay time constants are shown in Fig. S15. We find the frequency of electronic coherence to be 150 cm^{-1} with a decay time constant of 105 fs. Clearly, the electronic coherence lasts for two oscillation periods and completely disappears after 500 fs. In addition, the vibrational coherences show the frequencies of 65, 165 and 204 cm^{-1}. The long-lived vibrational coherences with decay time constants of 2.1 ps, 523 fs, 1.6 ps and with resolved phases generate the resonant beatings in the traces of the cross peaks in the 2D spectra.

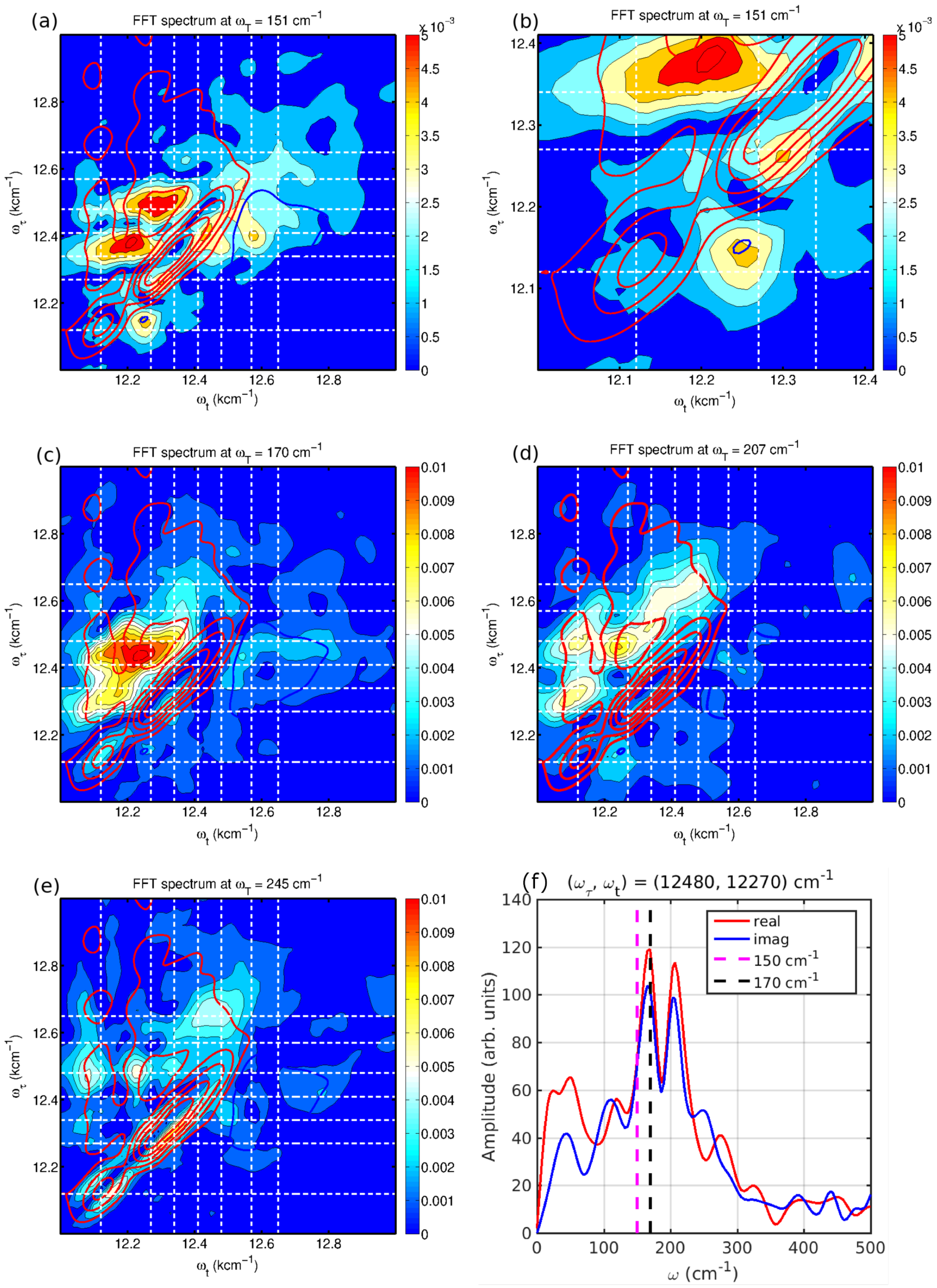

Fig. S8. (a) 2D power spectrum at the frequency $\omega_T = 151$ cm^{-1}, which is obtained by a Fourier transform of the 3D residuals after a global fitting approach. (b) Magnified view of the peaks associated to excitons 1 and 2. 2D vibrational patterns at the frequencies ω_T =170, 207 and 245 cm^{-1} are shown in (c), (d) and (e), respectively. (f) Power spectrum of the residual at (ω_τ, ω_t) = (12480, 12270) cm^{-1}.

Next, we describe the procedure of separating out electronic coherence at 50, 80 and 150 K. For this, we repeat the same procedure, the filtered resulting trace of the cross peak of the transition between exciton 1 and 2 is shown as black circles in Fig. S16. The obtained trace after fitting by the curve fitting toolbox is shown as red solid line. The boundaries of 95% are marked by blue dashed lines in Fig. S16. By this, the electronic coherence at 50 K is identified and shown as red solid line in Fig. S17.

Moreover, the polished trace of the cross peak between exciton 1 and 2 at 80 K is plotted as black circles in Fig. S18. The obtained fit is plotted as red solid line. The individual component of oscillation is shown in Fig. S19. It shows a decay time

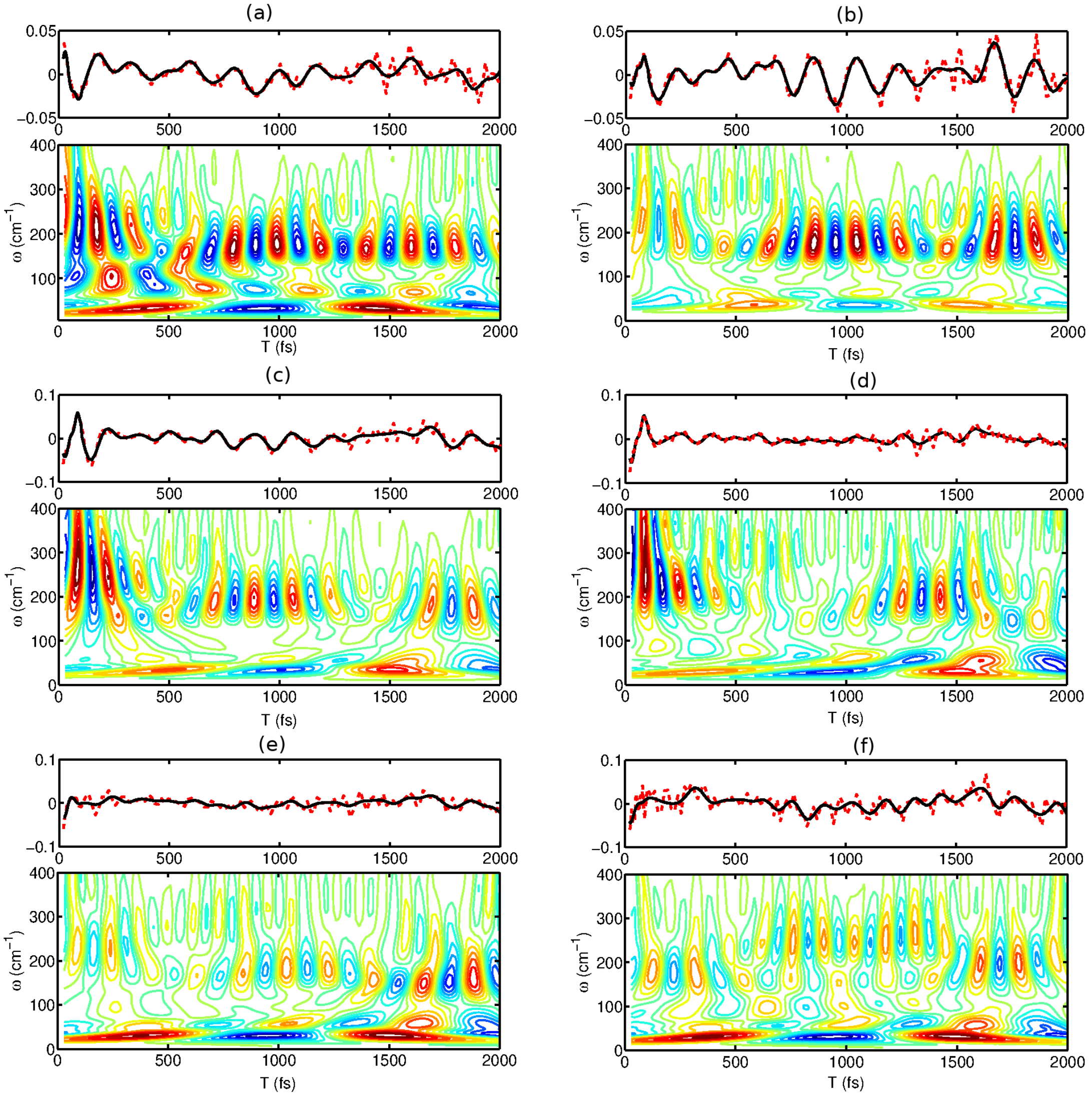

Fig. S9. Wavelet analysis of the beatings of the vibrations. (a) Residuals and vibrational dynamics of the cross peak at (ω_τ, ω_t) = (12270, 12120) cm^{-1}, and, correspondingly, in (b) to (f) for the cross peaks at (12410, 12120), (12480, 12120), (12570, 12120), (12650, 12120) and (12340, 12270) cm^{-1}, respectively.

constant of the electronic coherence of 81 fs at 80 K. In addition, the trace of the cross peak at 150 K and the obtained curve fitting results are shown in Fig. S20. The retrieved electronic coherence yields a decay time constant of 45 fs, which is shown in Fig. S21.

X. Dynamical electronic coherence of higher excitonic states

In the previous section, we have described the details to identify electronic coherence from the time trace of cross peaks associated to the transitions between two lowest-lying excitons. Here, we demonstrate the process how to extract the lifetime of electronic coherences of higher excitonic states. To monitor this electronic coherence, we examine the dynamics of the ESA peaks in the 2D spectra at 20 K. First, we extract the time trace of the ESA peak from the second exciton, which is marked as "A" in Fig. 4 of the main text. We plot the filtered trace as black circles in Fig. S22. The obtained fitting curve is plotted as red solid line with R-square $\geq$0.8. The boundaries of confidence (95%) are shown as blue dashed lines. We then analyze the oscillatory information by a Fourier transform, the result of which can be used as the initial values entering the fitting toolbox. The resolved oscillations occur with frequencies of 33, 117, 168, 219, 252 and 320 cm^{-1}, respectively. The power spectrum of the resolved cross peaks are shown in Fig. S23(a). We choose the starting values of the frequencies for the fitting tool from the Fourier transform and choose an identical decay time constant of 500 fs. These frequencies are adjusted during the fitting procedure which yields the optimal frequencies of 33, 112, 173, 206, 255 and 307 cm^{-1}. Among them, the strongest component of 206 cm^{-1} matches the energy gap between exciton 2 and 5. Based on our theoretical work (details of the calculations are presented in Sec. XII), we demonstrate that this component reflects the electronic coherence between exciton 2 and 5. We show

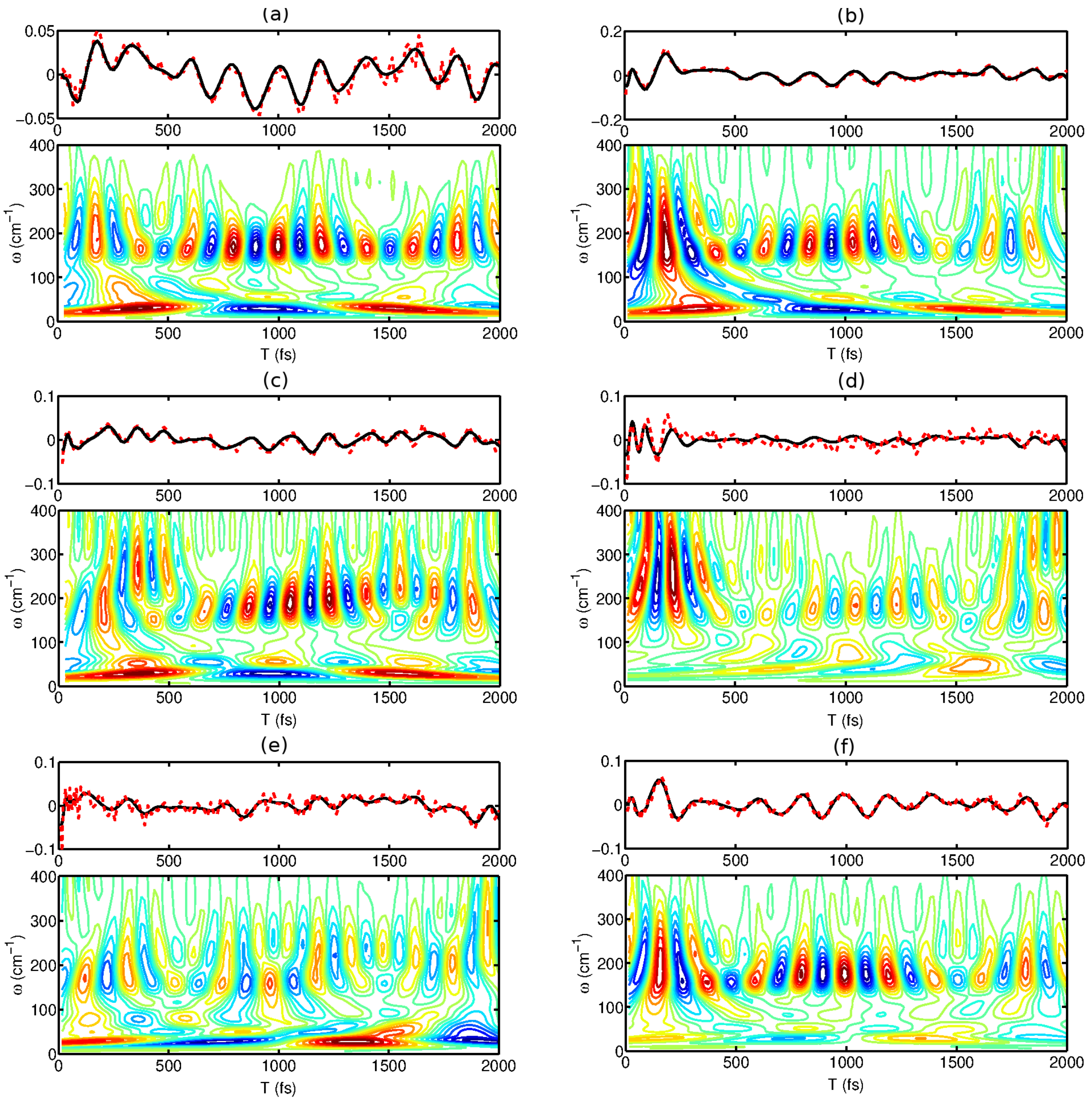

Fig. S10. Same as Fig. S9, but (a)-(f) of the cross peaks retrieved from (12410, 12270), (12480, 12270), (12570, 12270), (12650, 12270), (12410, 12340) and (12480, 12340) cm^{-1}, respectively.

it as red solid line in Fig. S23(b). It yields a decay time constant of 91 fs. Repeating the same step, we study the time trace of the ESA peak of exciton 5, which is marked by the label "B" in Fig. 4 of the main text. In Fig. S24, we show the polished trace as black circles. The starting values of the frequencies for the fitting tool are taken from a Fourier transform and are set to 25, 62, 167, 210, 250 and 305 cm^{-1}. In addition, we set the initial values of decay time constants for each component to be 500 fs. The resulting time trace after the fitting procedure is shown as red solid line in Fig. S24 with R-square $\geq$0.8. From this, we conclude that the strongest component shows the frequency of 210 cm^{-1} with a decay time constant of 91 fs. This is shown as blue solid line in Fig. S23(b). Fig. S23(b) clearly shows anti-correlated oscillations in both time traces, although the phase is not perfectly anti-correlated. We believe this to be caused by the noise in the ESA peaks.

Next, we need to examine the coherent dynamics of exciton 3 and 7. From the basis transformation, we know that excitons 3 and 7 are mainly composed of contributions of pigments 1 and 2, where a strong electronic coupling occurs. First, we extract the time trace of the ESA peak "C" in Fig. 4 in the main text. Repeating the same procedure, the polished trace is obtained and plotted as black circles in Fig. S25. To set the starting values for the fitting procedure, a Fourier transform is performed to identify the oscillation frequencies. In addition, we set the starting decay time constants of each component again to 500 fs. With the help of the curve fitting toolbox, we obtain the fitting trace and plot it as red solid line in Fig. S25. Moreover, the filtered trace and the fitting result of ESA peak "D" are shown as black circles and red solid line in Fig. S26. By this, we identify the strongest component at 310 cm^{-1} in Fig. S27, which agrees with the energy gap between exciton 3 and 7. By this calculation, we demonstrate clear evidence of the electronic coherence, with the decay time constants of 59 fs (blue trace) and 34 fs (red trace) for the ESA peaks "C" and "D".

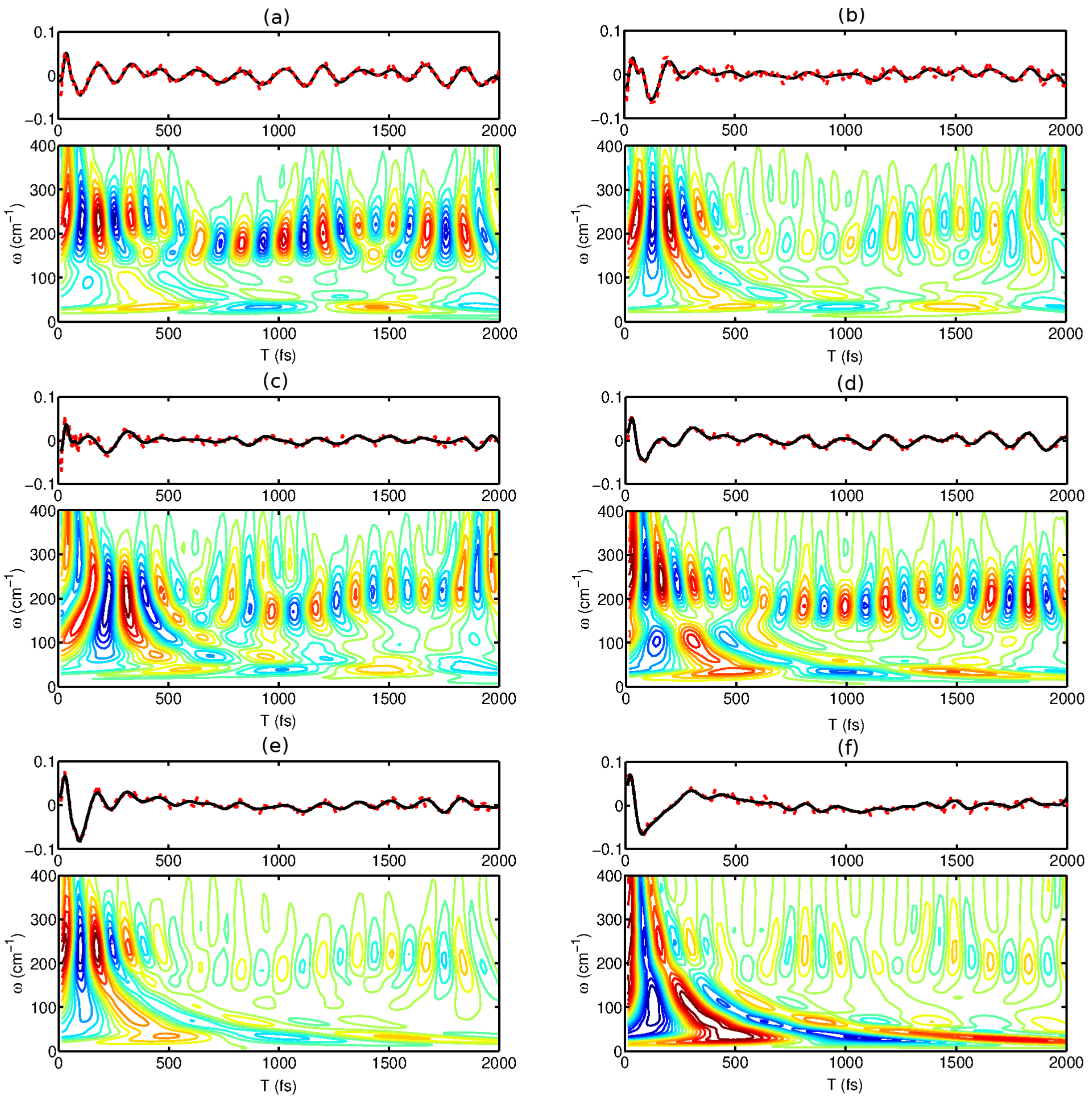

Fig. S11. Same as Fig. S9, but (a)-(f) of the cross peaks retrieved from (12570, 12340), (12650, 12340), (12480, 12410), (12570, 12410), (12650, 12410) and (12570, 12480) cm^{-1}, respectively.

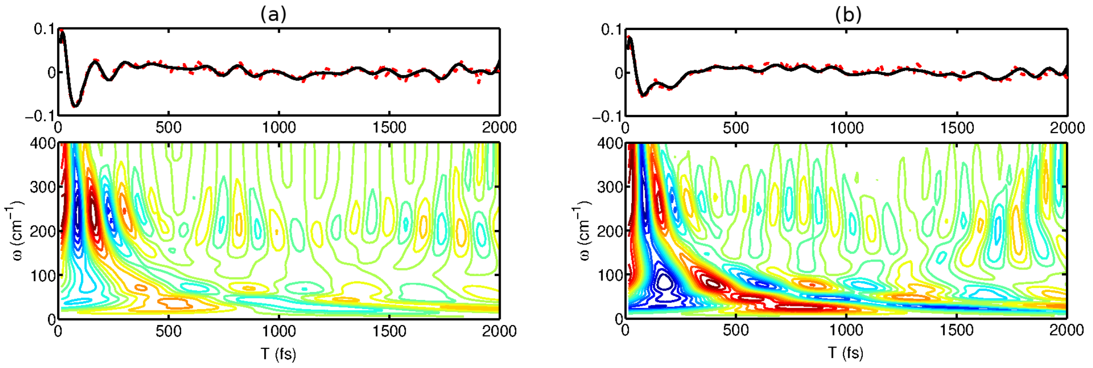

Fig. S12. Same as Fig. S9, but (a),(b) of the cross peaks retrieved from (12650, 12480) and (12650, 12570) cm^{-1}, respectively.

XI. Optical dephasing vs. electronic decoherence

As discussed in the main paper, there are two different processes related to electronic coherence. One is optical coherence between the ground and the excited states. A measure for this is the width in the anti-diagonal direction of a diagonal peak of

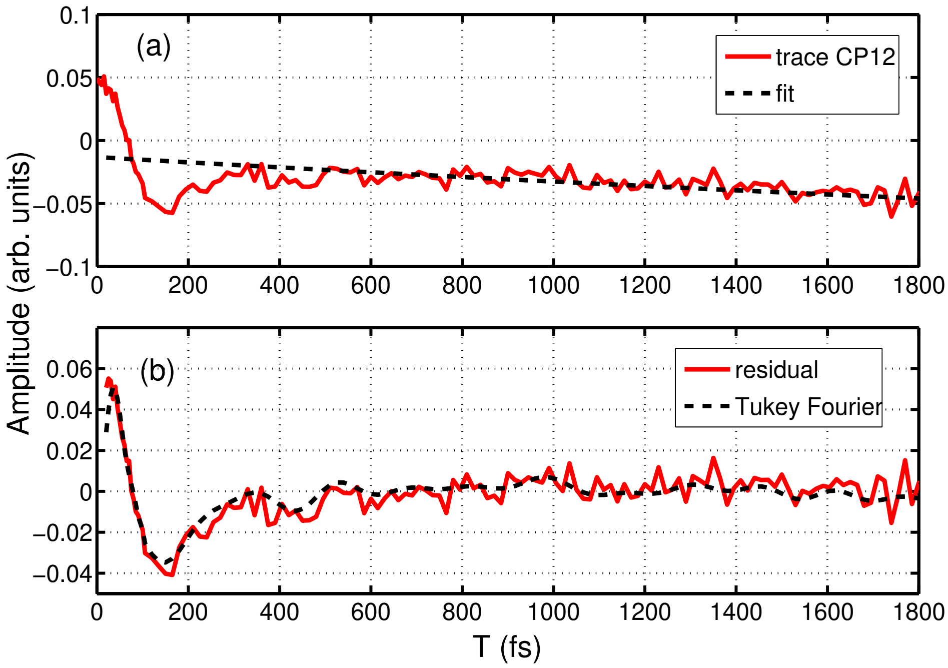

Fig. S13. (a) Trace (red solid line) of raw data of the cross peak between exciton 1 and 2 at (ω_τ, ω_t) = (12120, 12270) cm^{-1} at 20 K. The kinetics can be removed by exponential fitting (black dashed line). (b) Residual (red solid line) after removing the kinetics. The high-frequency jitter is removed by a Tukey window Fourier transform and the polished trace is plotted as black dashed line in (b).

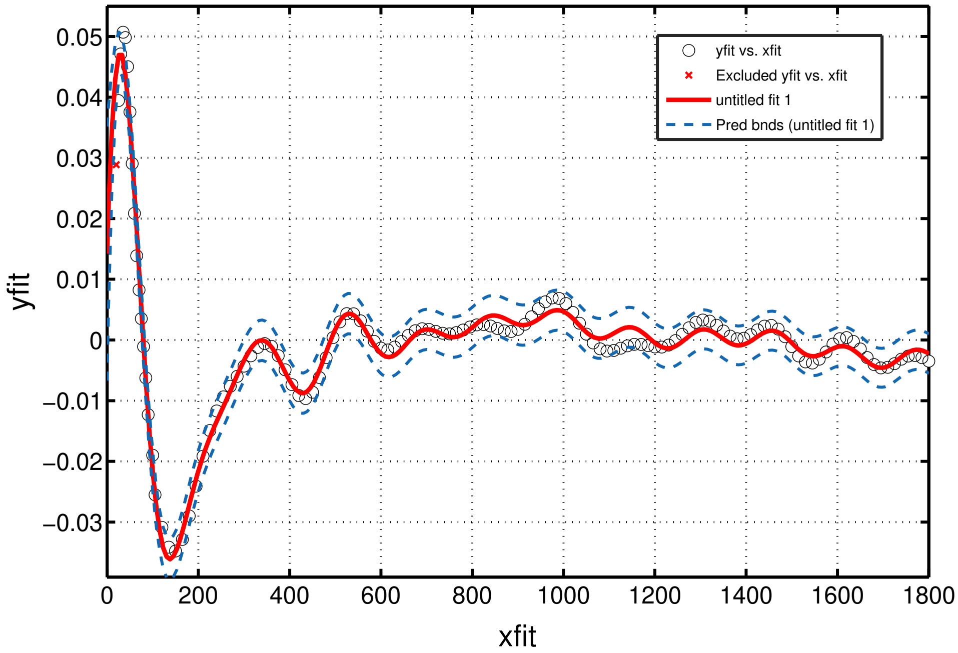

Fig. S14. The fitting results of the trace of the cross peak between exciton 1 and 2 obtained by the curve fitting toolbox. The trace and the obtained fitting curve are shown as black circles and red solid line, respectively. The boundaries of confidence (95%) are plotted as blue dashed lines.

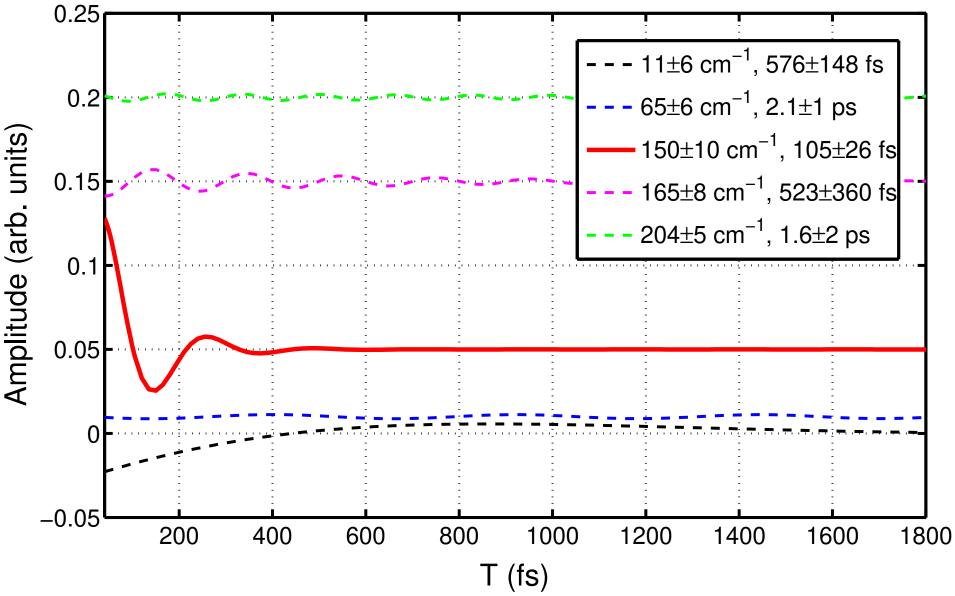

Fig. S15. Electronic and vibrational coherences resolved by the curve fitting processes. The identified oscillations occur with frequencies of 11, 65, 150, 165 and 204 cm^{-1} with related decay time constants as indicated. Each component is shifted vertically from zero to show them separately.

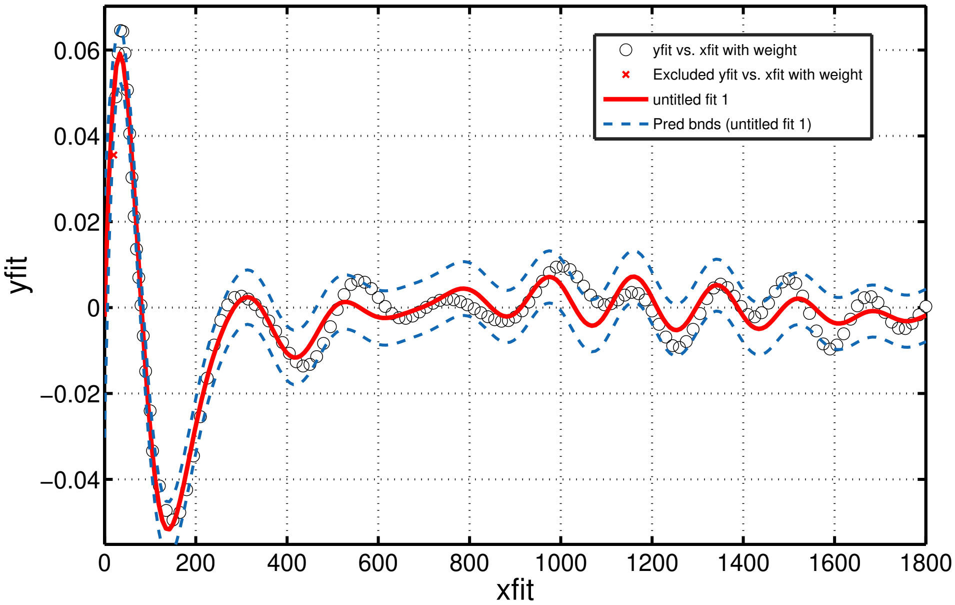

Fig. S16. Same as in Fig. S14, but for 50 K. The initial time point is excluded to achieve a converged fit.

a 2D spectrum at zero waiting time. A second process is electronic coherence between two exciton states between which a transfer occurs. This is characterized by the decay time constant of the associated cross peak in the 2D spectrum as a function of the waiting time. Both processes are different, but related to each other.

We show the direct comparison of the associated time constants of the optical dephasing and the electronic decoherence at different temperatures in Fig. 1(j) of the main text. Based on our measurements (including the errorbars), we find that both timescales are comparable in the region from 20 K to 300 K, but not identical. It is known that for a complete Markovian dynamics (i.e., a high temperature), both time constants show the same scales. In our case, the agreement of the optical dephasing and the decoherence time constants indicate that non-Markovian effects in the FMO complex do not play a significant

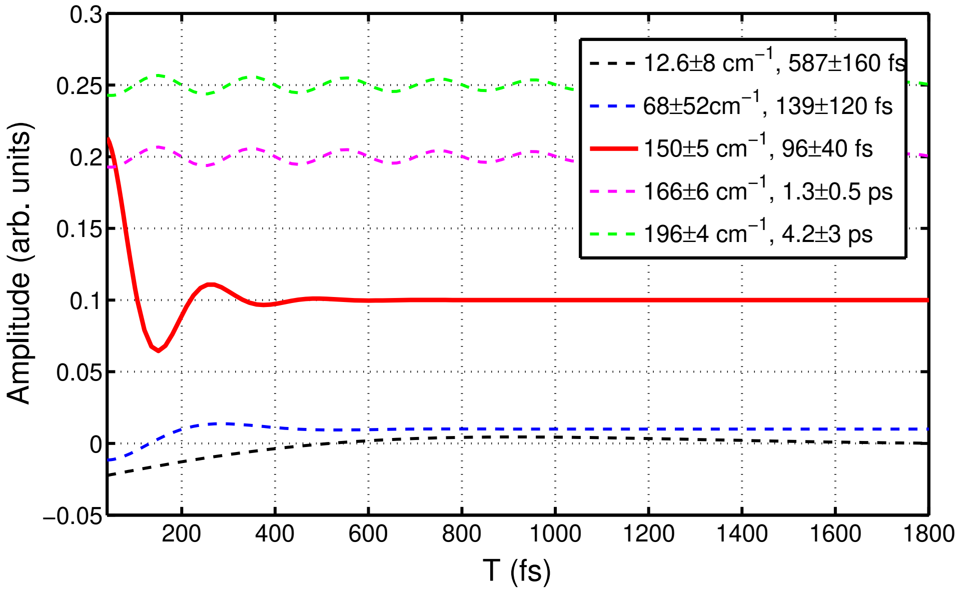

Fig. S17. Same as in Fig. S15, but for 50 K. The electronic coherence (red solid line) shows the frequency of 150 cm^{-1} with a decay time constant of 96 fs. Each of the component is shifted vertically from zero to distinguish the oscillations.

role even at low temperature 20 K.

XII. Site basis to Exciton basis

The one-exciton Hamiltonian of the FMO protein reads

$$H_S = \begin{pmatrix} 280 & -106 & 8 & -5 & 6 & -8 & -4 \\ -106 & 420 & 28 & 6 & 2 & 13 & 1 \\ 8 & 28 & 35 & -62 & -1 & -9 & 17 \\ -5 & 6 & -62 & 180 & -70 & -19 & -57 \\ 6 & 2 & -1 & -70 & 325 & 40 & -2 \\ -8 & 13 & -9 & -19 & 40 & 365 & 32 \\ -4 & 1 & 17 & -57 & -2 & 32 & 265 \end{pmatrix} + \lambda_S \mathbf{I} \qquad \text{[S6]}$$

in units of cm^{-1} in the site representation, where we have added an energy shift λ_S (λ_S=12205cm^{-1}, 12210cm^{-1}, 12240cm^{-1} at temperatures of 50K, 80K, 150K, respectively) to the diagonal elements of the exciton Hamiltonian to match the frequency range of the absorption spectra. The exciton states of the FMO complex are then obtained by diagonalizing Eq. (S6). The squares of the eigenvector elements of the seven exciton states with exciton index in ascending energy order are tabulated in Tab. S1.

Table S1. Square of the eigenvector elements of the exciton Hamiltonian in Eq. (S6) in the exciton basis with the exciton index in the order of ascending energy. The negative sign (-) denotes that the corresponding eigenvector element is negative and bold numbers label the dominant site contribution to the exciton state.

	Bchl 1	Bchl 2	Bchl 3	Bchl 4	Bchl 5	Bchl 6	Bchl 7
Exciton 1	(-)0.0028	(-)0.0068	**0.8432**	**0.1390**	0.0068	0.0011	0.0003
Exciton 2	0.0068	0.0024	(-)**0.1117**	**0.5800**	**0.0990**	(-)0.0049	**0.1953**
Exciton 3	**0.7668**	**0.2103**	0.0111	(-)0.0003	(-)0.0083	0.0021	(-)0.0011
Exciton 4	0.0000	(-)0.0000	(-)0.0238	**0.1154**	0.0697	0.0683	(-)**0.7227**
Exciton 5	0.0013	0.0095	0.0067	(-)0.0723	**0.5013**	(-)**0.3995**	(-)0.0093
Exciton 6	0.0071	(-)0.0145	0.0013	(-)0.0930	**0.3136**	**0.5009**	0.0696
Exciton 7	**0.2151**	(-)**0.7564**	(-)0.0021	0.0001	(-)0.0013	(-)0.0232	(-)0.0017

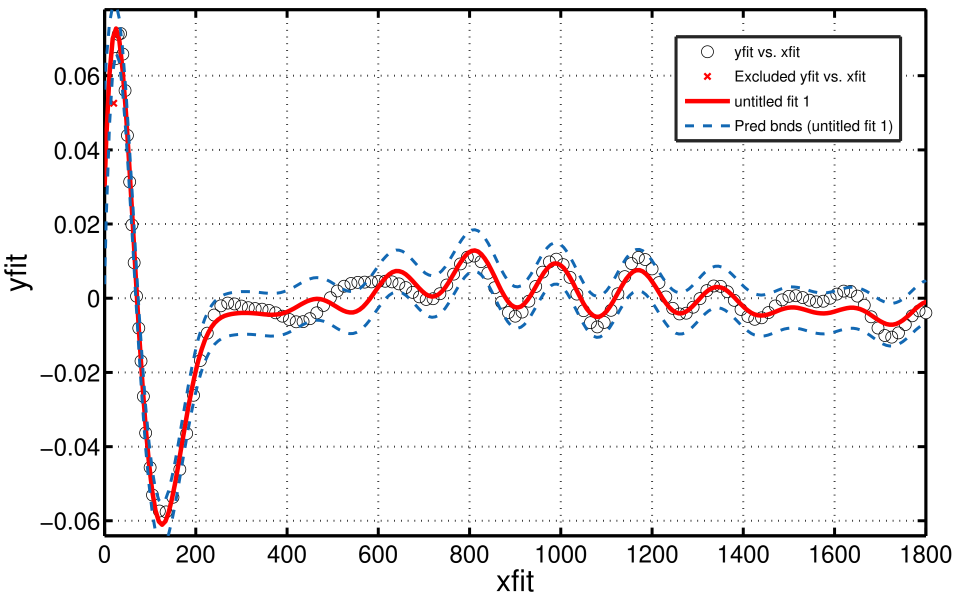

Fig. S18. Same as in Fig. S14, but for 80 K.

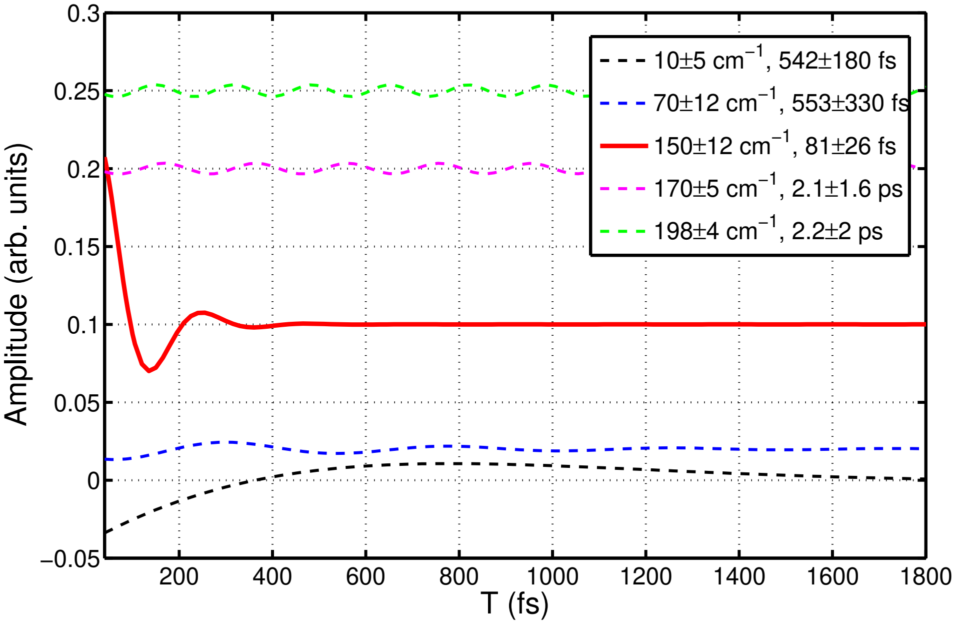

Fig. S19. Same as in Fig. S15, but for 80 K. Electronic coherence (red solid line) shows the decay time constant of 81 fs at a frequency of 150 cm^{-1}.

XIII. Calculation of absorption spectra

The dipole operator that governs the photon-exciton conversion is given by $\boldsymbol{\mu} = \boldsymbol{\mu}^+ + \boldsymbol{\mu}^-$ with

$$\boldsymbol{\mu}^+ = \sum_{m=1}^{N} \mu_m |m\rangle\langle g| \,,$$

$$\boldsymbol{\mu}^- = \sum_{m=1}^{N} \mu_m |g\rangle\langle m| \,, \qquad [S7]$$

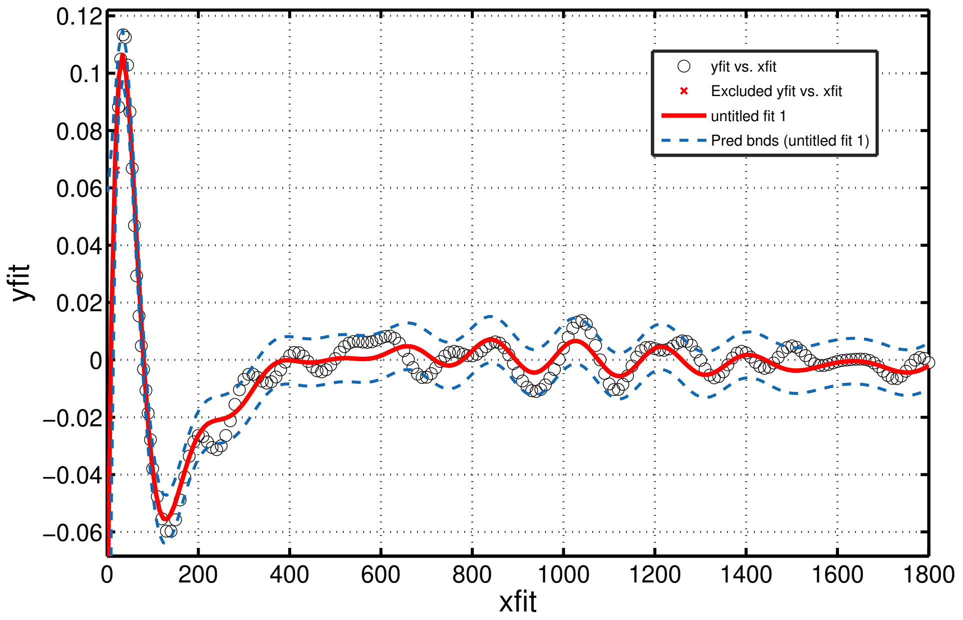

Fig. S20. Same as in Fig. S14, but for 150 K.

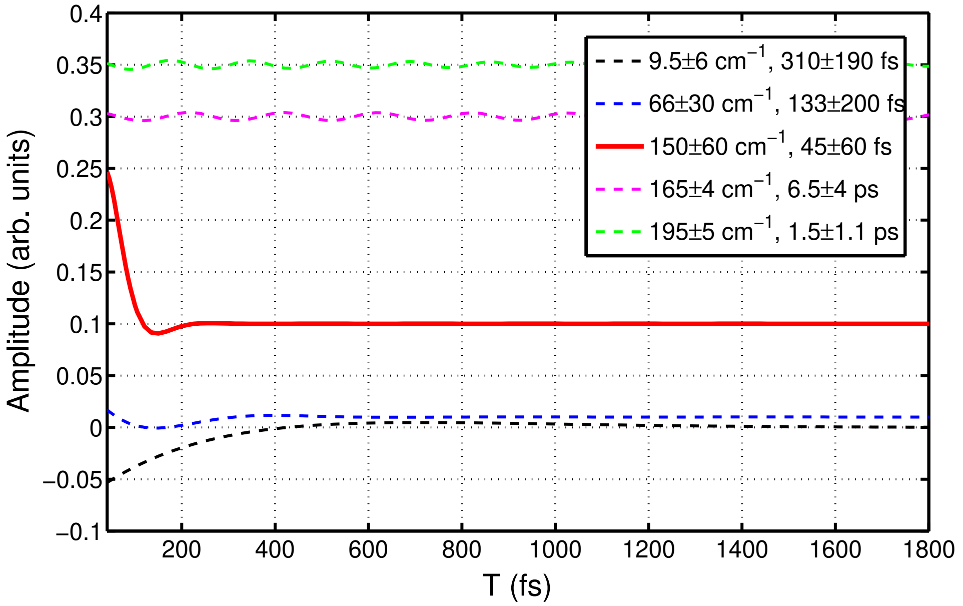

Fig. S21. Same as in Fig. S15, but for 150 K. The component of the electronic coherence at 150 cm^{-1} yields a decay time constant of 45 fs.

where μ_m is the transition dipole moment of the mth site, which are extracted from crystallographic data of the FMO complex (data from 3ENI.pdb). The absorption spectrum is then calculated as

$$I(\omega) = \left\langle \int_0^\infty dt e^{i\omega t} \mathrm{tr}(\boldsymbol{\mu}(t)\boldsymbol{\mu}(0)\rho_g) \right\rangle_{\mathrm{rot}} , \qquad \text{[S8]}$$

where $\rho_g = |g\rangle\langle g|$ and a δ-shaped laser pulse is assumed. $\langle\cdot\rangle_{\mathrm{rot}}$ denotes the rotational average of the molecules with respect to the laser direction. For linear spectroscopy, the rotational averaging can be easily described by considering three representative

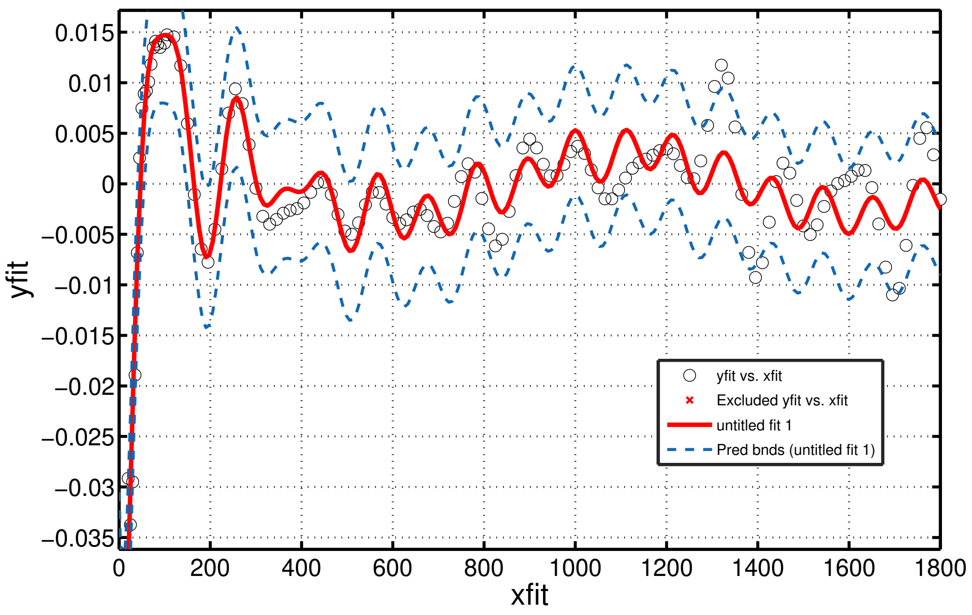

Fig. S22. Filtered time trace of the ESA peak “A" in Fig. 4 of the main text. The resulting fitting curve is plotted as red solid line.

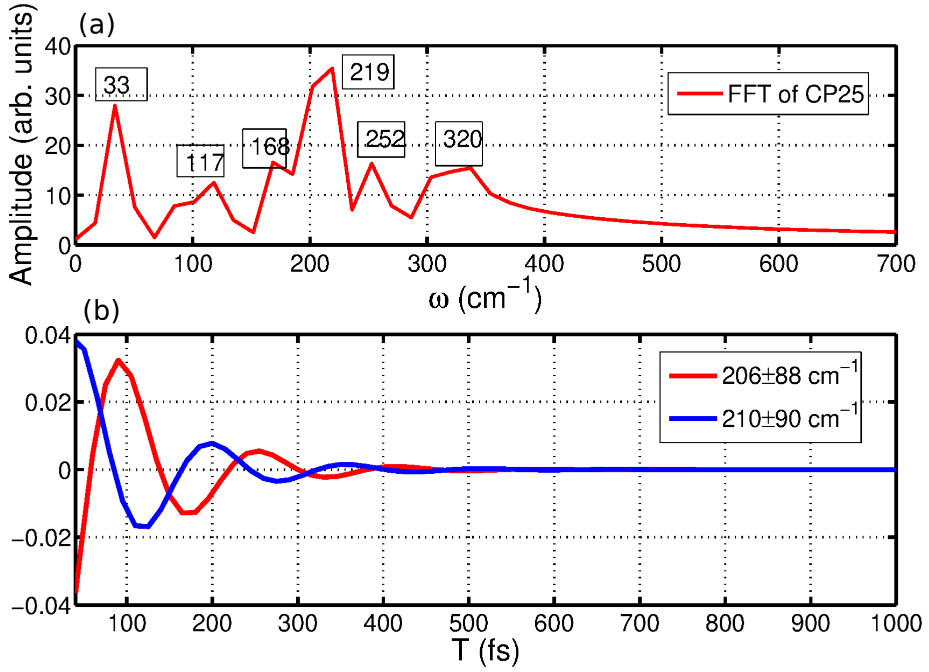

Fig. S23. (a) Power spectrum of the ESA peak “A” in Fig. 4 of the main text. The identified frequencies are marked and indicated at the peak position. (b) Strongest frequency components of 206 (ESA peak A) and 210 (ESA peak B) cm^{-1}. Both show the same decay time constant of ~91 fs.

electric fields along the Cartesian unit vectors (9)

$$\mathbf{e}_1 = \{1,0,0\}, \quad \mathbf{e}_2 = \{0,1,0\}, \quad \mathbf{e}_3 = \{0,0,1\}\,. \qquad \text{[S9]}$$

In addition, the static disorder is modelled by sampling the site energies ϵ_m from uncorrelated Gaussian distributions with a standard deviation σ_m for each Bchl. All absorption spectra were averaged over 1000 realizations of site energies. By fitting the experimental absorption spectra at different temperatures, we find the optimal set of σ_m: $\sigma_1 = 50\text{cm}^{-1}$, $\sigma_2 = 45\text{cm}^{-1}$,

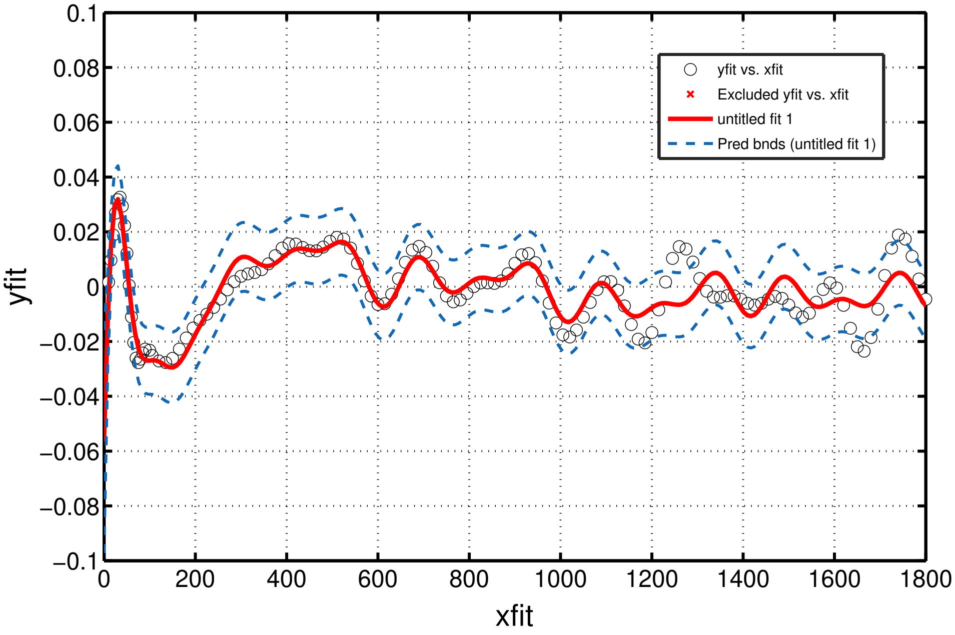

Fig. S24. Curve fitting results of the ESA peak "B" in Fig. 4 of the main text.

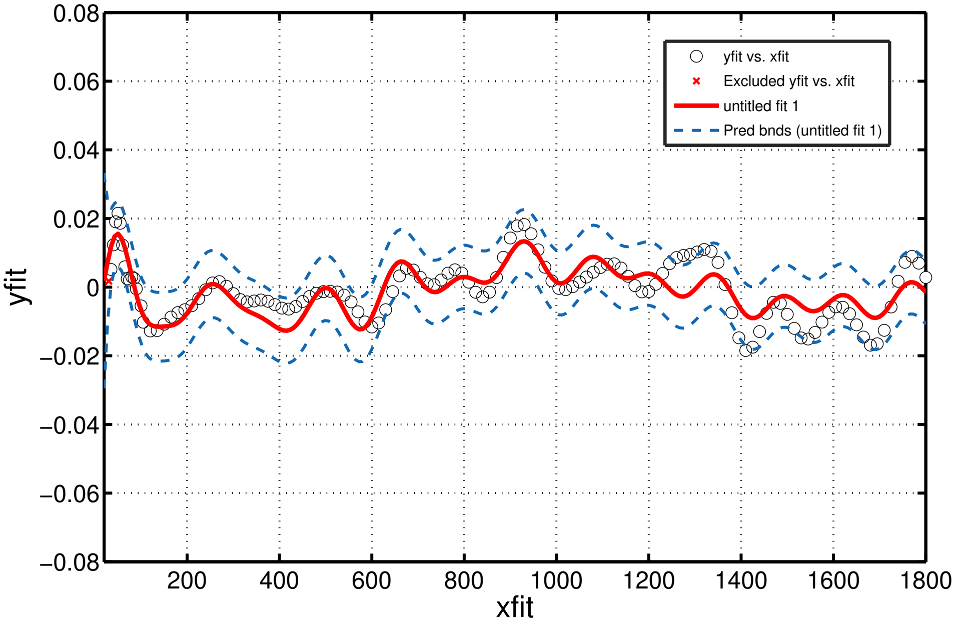

Fig. S25. Curve fitting results of the ESA peak "C" in Fig. 4 of the main text.

$\sigma_3 = 30\text{cm}^{-1}$, $\sigma_4 = 50\text{cm}^{-1}$, $\sigma_5 = 50\text{cm}^{-1}$, $\sigma_6 = 45\text{cm}^{-1}$, $\sigma_7 = 55\text{cm}^{-1}$. The calculated absorption spectra and comparisons with experimental ones at 77K and 300K are shown in Fig. S28.

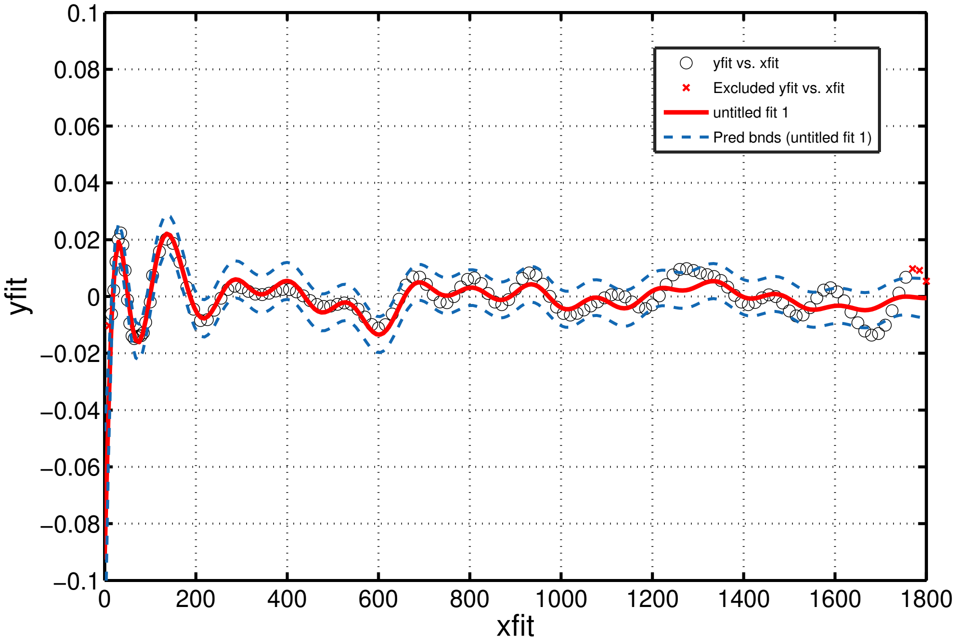

Fig. S26. Curve fitting results of the ESA peak "D" in Fig. 4 of the main text.

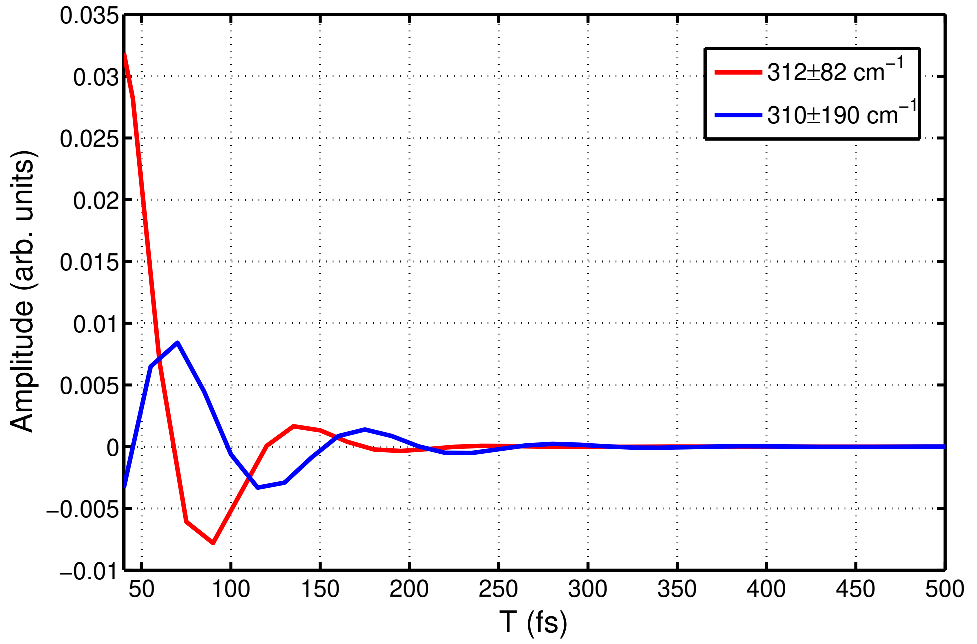

Fig. S27. Electronic coherence between exciton 3 (blue) and 7 (red solid line). We extract the decay time constants of two coherences as 59 and 34 fs, respectively.

XIV. Calculation of 2D electronic spectra

The simulation of 2D electronic spectra involves the calculation of the third-order response function

$$S^{(3)}(t,T,\tau) = \left(\frac{i}{\hbar}\right)^3 \Theta(t)\Theta(T)\Theta(\tau)\mathrm{tr}\left\{\boldsymbol{\mu}(t+T+\tau)\left[\boldsymbol{\mu}(T+\tau),\left[\boldsymbol{\mu}(\tau),\left[\boldsymbol{\mu}(0),\rho_g\right]\right]\right]\right\}\ . \qquad \text{[S10]}$$

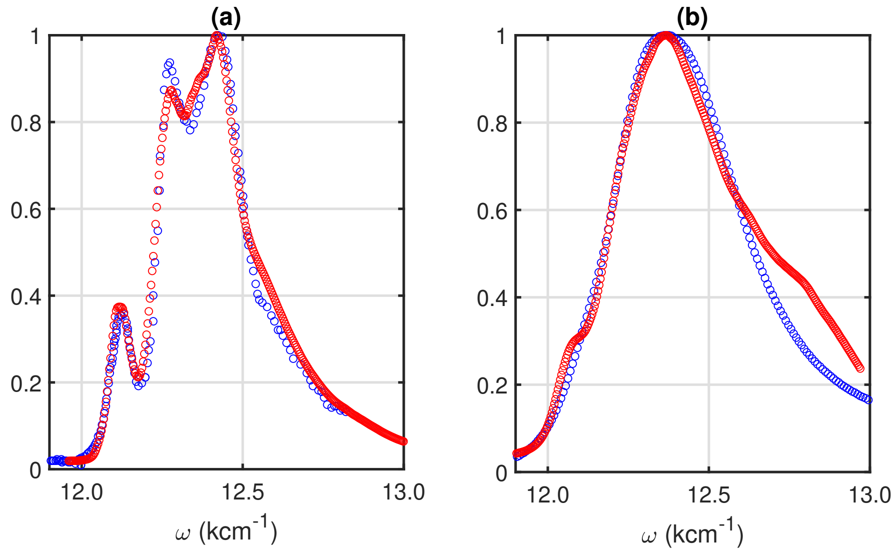

Fig. S28. Calculated absorption spectra (red circular dots) of FMO complex at 77 K (a) and 300 K (b). The measured absorption are shown as blue circular dots.

Here, τ is the delay time between the second and the first pulse, T (the so-called population time) is the delay time between the third and the second pulse, and t is the detection time. To evaluate 2D electronic spectra, we need the so-called rephasing (RP) and non-rephasing (NR) contributions of the third-order response function, i.e., $S^{(3)}(t,T,\tau) = S^{(3)}_{\rm RP}(t,T,\tau) + S^{(3)}_{\rm NR}(t,T,\tau)$. Assuming the impulsive limit (the δ-shaped laser pulse), one obtains

$$I_{\rm RP}(\omega_t,T,\omega_\tau) = \int_{-\infty}^{\infty} d\tau \int_{-\infty}^{\infty} dt e^{i\omega_t t - i\omega_\tau \tau} S^{(3)}_{\rm RP}(t,T,\tau), \quad [S11]$$

$$I_{\rm NR}(\omega_t,T,\omega_\tau) = \int_{-\infty}^{\infty} d\tau \int_{-\infty}^{\infty} dt e^{i\omega_t t + i\omega_\tau \tau} S^{(3)}_{\rm NR}(t,T,\tau). \quad [S12]$$

The total 2D signal is the sum of the two

$$I(\omega_t,T,\omega_\tau) = I_{\rm RP}(\omega_t,T,\omega_\tau) + I_{\rm NR}(\omega_t,T,\omega_\tau)\,. \quad [S13]$$

The rephasing and the non-rephasing parts include the contributions of six different Liouville pathways, which correspond to the stimulated emission, the ground-state bleach (GSB) and the excited state absorption (ESA) (4). In order to account for the contribution of the excited state absorption, we have extended the one-exciton Hamiltonian to the double-excited manifold. The two-exciton Hamiltonian has $[N(N-1)/2]^2$ elements which can be constructed from the single-exciton Hamiltonian as described in Ref. (10). To calculate 2D spectra, we propagate both the density matrix and the auxiliary matrices by the time non-local quantum master equation according to the six Liouville pathways.

In ensemble experiments, the molecules are randomly oriented with respect to the laser direction and one thus needs to compute rotational averages $\langle \mu_i^2 \mu_j^2 \rangle_{\rm rot}$. In this work, the Bchl molecules in the FMO complex were kept fixed, we perform the rotational averages by sampling ten laser polarization vectors following the procedures described in Ref.(9). Following this prescription, we calculate 2D electronic spectra of the FMO complex at different temperatures and show them in Fig. S29. Despite the complexity in modelling the spectra, we found that the overall agreement with experiments is good (see Fig. S2, S3 and S4). The major difference comes from the higher-energy excitonic states, which is due to the impulsive limit approximation employed in the simulation. It is noted that laser profile used in the experiments, on the other hand, is blue-shifted as compared to the frequency range of the absorption spectrum of the FMO complex (see Fig. S1).

To efficiently compute the evolution of a specific peak as a function of the waiting time, we have combined the equation of motion-phase matching approach (EOM-PMA) (11) with the scheme proposed in Ref. (12). By simulating 2D electronic spectra as four-wave-mixing signals generated by two femtosecond pulses and two one-sided continuous-wave pulses, this method can directly yield calculated 2D spectra at a specific excitation frequency ω_τ and detection frequency ω_t as a function of the waiting time T. We analyze the time evolution of the cross peak between exciton 1 and 2 (CP21) by this method, and plot the traces at different temperatures (50, 80 and 150 K) in Fig. 5 of main text. The residuals are obtained by an exponential fit, and the subsequent electronic and vibrational coherences are extracted by curve fitting toolbox in Matlab. We present the dynamics of electronic and vibrational coherences in Fig. S30. At 50K (Fig. S30(a)), one observes that while the electronic coherence lasts for two periods and completely disappears at 500fs, the vibrational coherence sustains for more than 1 ps. It is further found that the electronic coherences (150 cm^{-1}) are dramatically damped with the increase of the temperature, and the vibrational coherences (180 cm^{-1}), on the other hand, are still robust when increasing the temperature, as illustrated in Fig. S30(b) and (c).

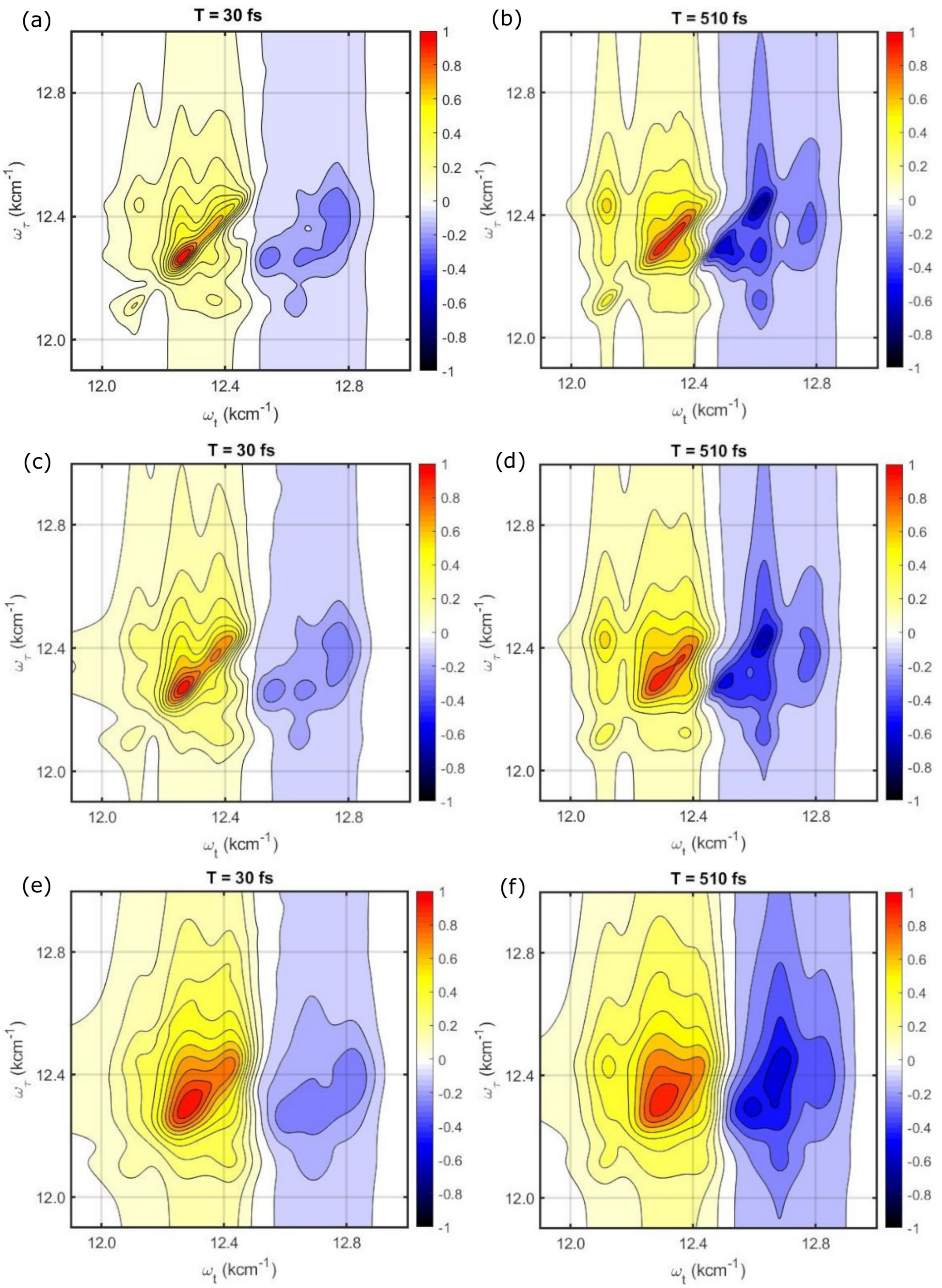

Fig. S29. Calculated 2D electronic spectra of the FMO complex at 50 (a and b), 80 (c and d) and 150 K (e and f). The waiting times are indicated at the top of each 2D spectrum.

XV. Theoretically retrieved electronic coherences

In this section, we examine the calculated lifetimes of the electronic coherences of the FMO complex at different temperatures. Due to the small scale of the simulation, a numerically exact approach, the hierarchy equation of motion (HEOM), is applicable to calculate the population dynamics of the FMO complex. The details of the HEOM method are described in Refs. (13, 14). To mimic the optical excitation in spectroscopy, we assume that the initial populations are equally distributed on each site of the FMO complex. Moreover, to compare with the previous works, we also calculate the population dynamics of the FMO

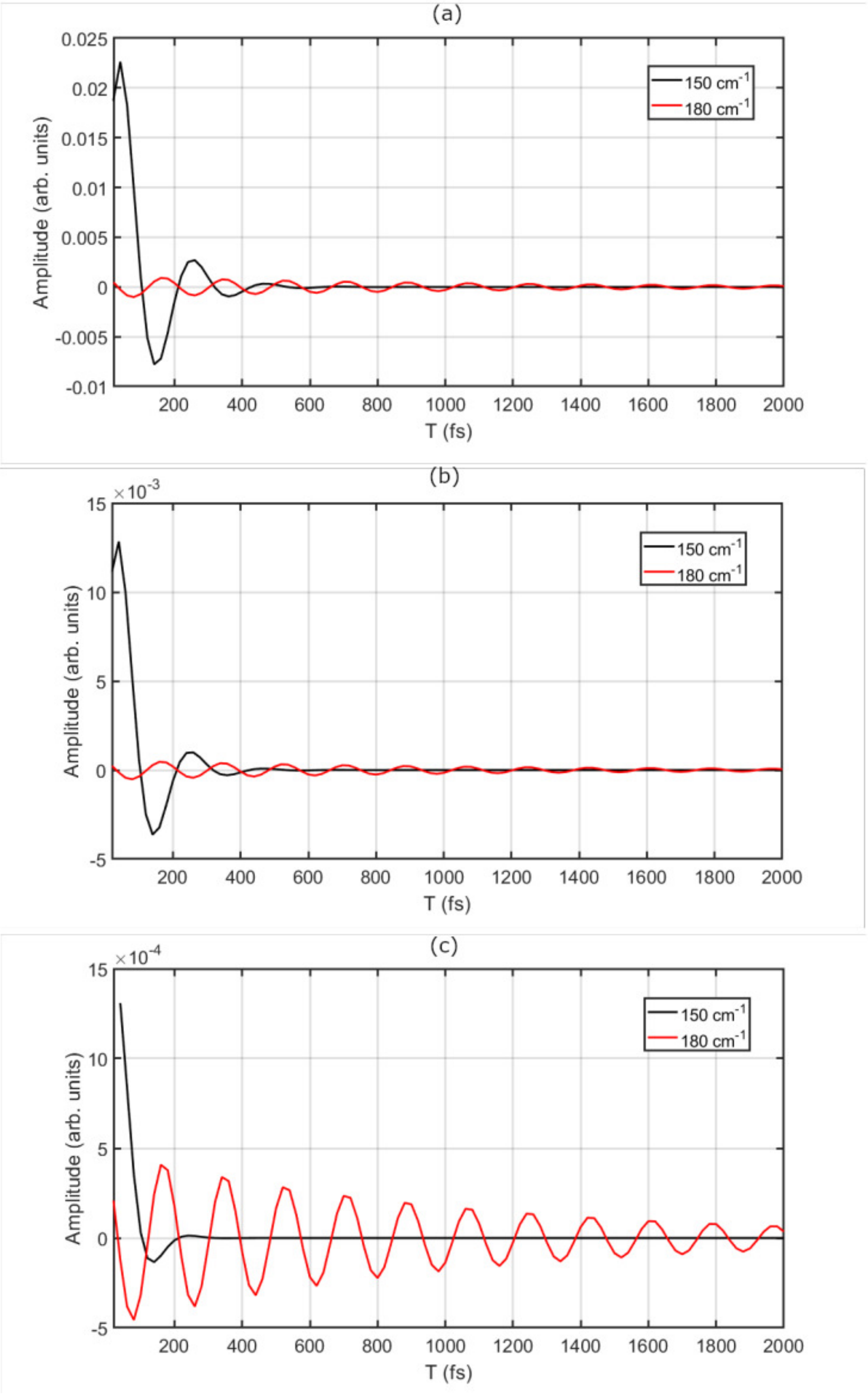

Fig. S30. Electronic (red solid lines) and vibrational coherences (black solid lines) at 50 K (a), 80 K (b) and 150 K (c). The extracted lifetimes of the electronic coherences are 90±22 fs, 80±25 fs and 45±40 fs, respectively.

complex with an initial population located on site 1, that is, $\rho(t=0) = |1\rangle\langle 1|$. The resulting population dynamics of the FMO complex are shown in Figs. S31 to S36.

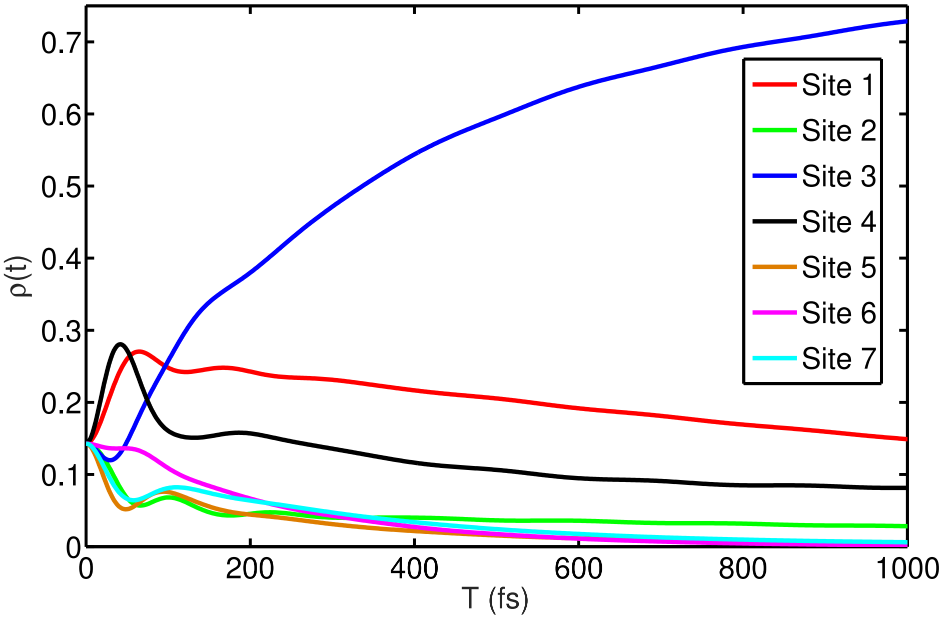

Fig. S31. Population dynamics of the seven sites of the FMO complex at 50 K. The initial populations are assumed to be equally distributed on the seven sites.

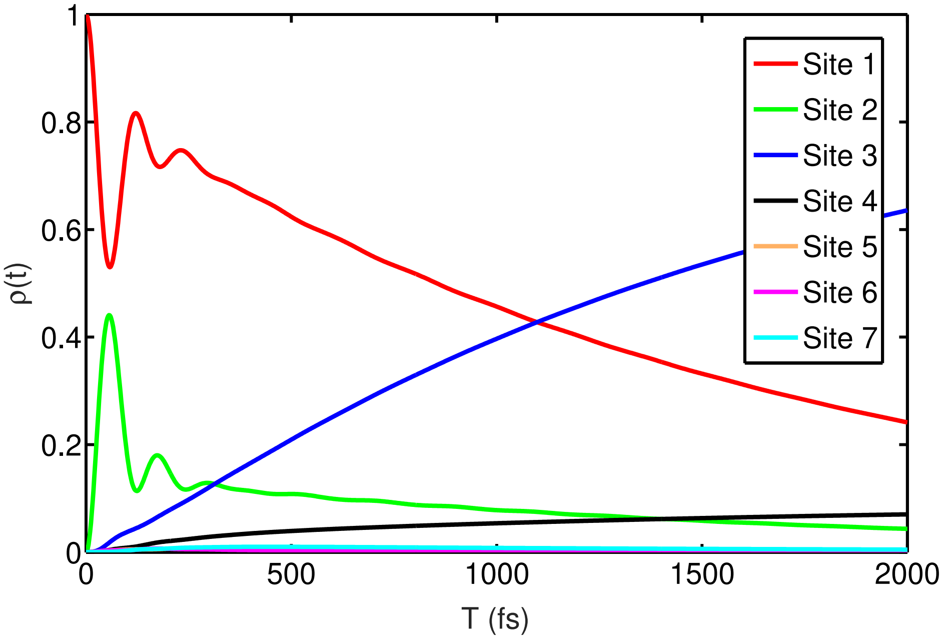

Fig. S32. Same as in Fig. S31, but for the initial state being fully localized on the site 1.

XVI. Quantum coherence calculated with a reorganization energy of 35 cm^{-1}

In this section, we calculate the 2D electronic spectra and examine the coherent dynamics of the lowest-energy exciton (the exciton 1) for the same model described in the main text, but with a smaller reorganization energy of 35 cm^{-1}. We extract the time evolution of the cross peak (CP21) from 2D electronic spectra and plot it as circular dotted red line in Fig. S37(a). To retrieve the coherent dynamics, we first remove the kinetics by fitting with two exponential functions, and then analyze the obtained residuals by the Eq. (S5). The retrieved electronic and vibrational coherences are shown in Fig. S37(b). The lifetimes

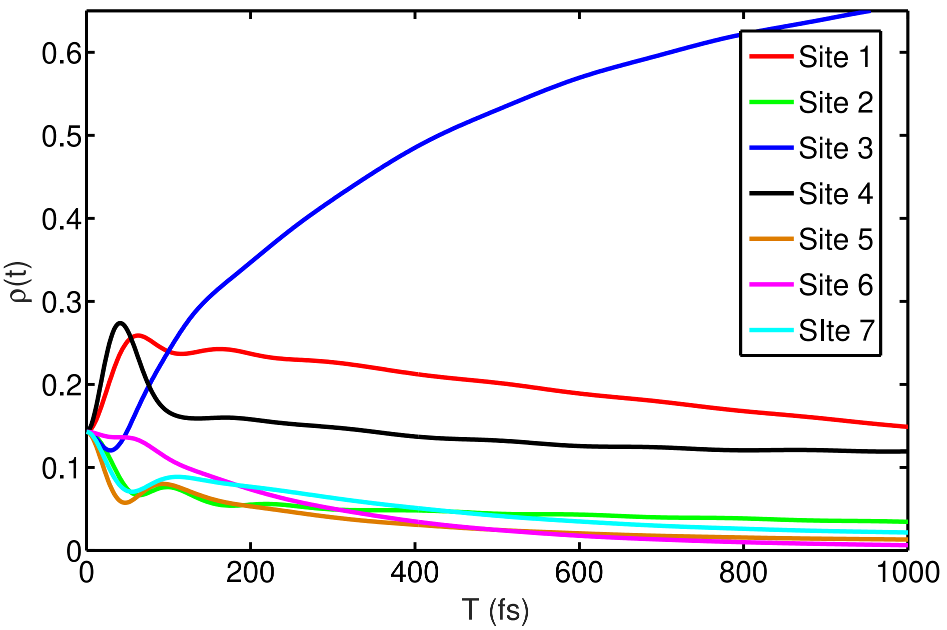

Fig. S33. Same as Fig. S31, but for 80 K. The initial populations are equally located on all seven sites.

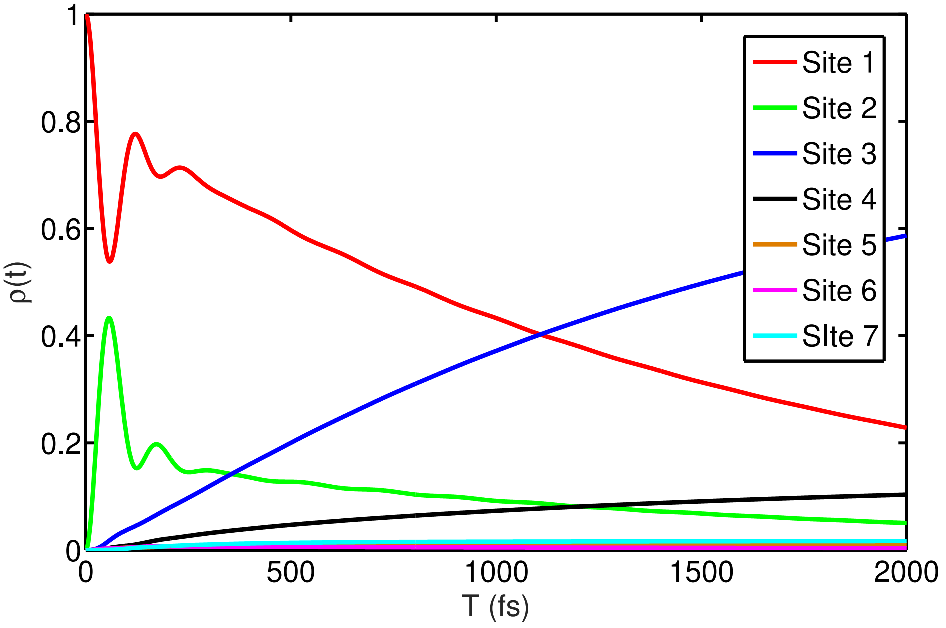

Fig. S34. Same as in Fig. S32, but for 80 K. The initial population is located on the site 1.

of the electronic and vibrational coherences are found to be 137 fs and 420 fs, respectively.

XVII. Origin of the beatings in the electronic dynamics

In this section, we investigate the origin of the beatings in the electronic dynamics as observed in the measurements. To this end, we consider a relatively strongly coupled dimer of pigment 3 and 4 of the FMO complex, which contributes to the exciton 1 and 2. The inter-pigment electronic coupling strength and the site-energy difference are -62 and 145 cm^{-1}, respectively. The system is coupled to a dissipative environment, the effect of which can be characterized by the spectral density. We employ the

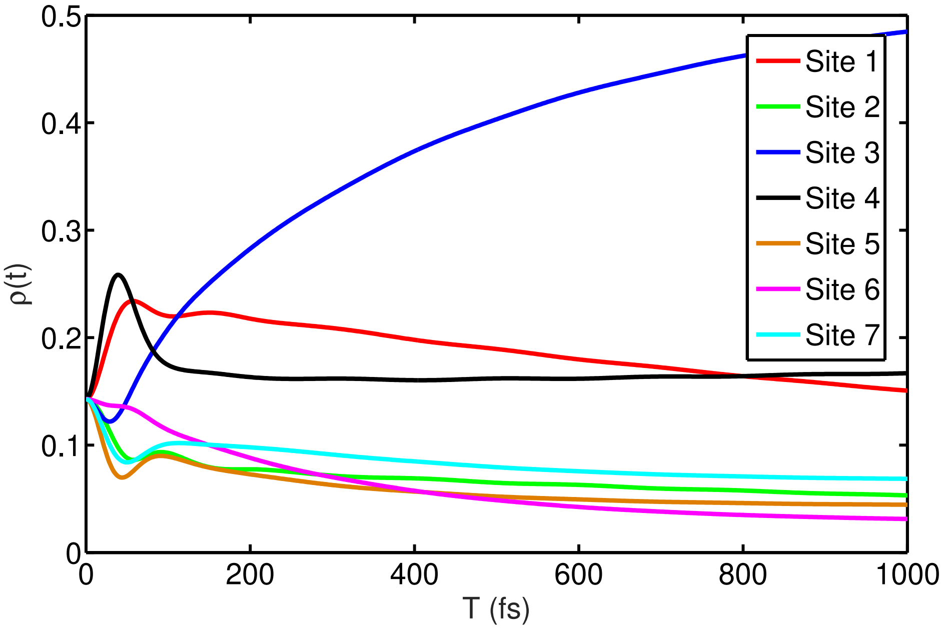

Fig. S35. Same as Fig. S31, but for 150 K.The initial population is equally located on all seven sites.

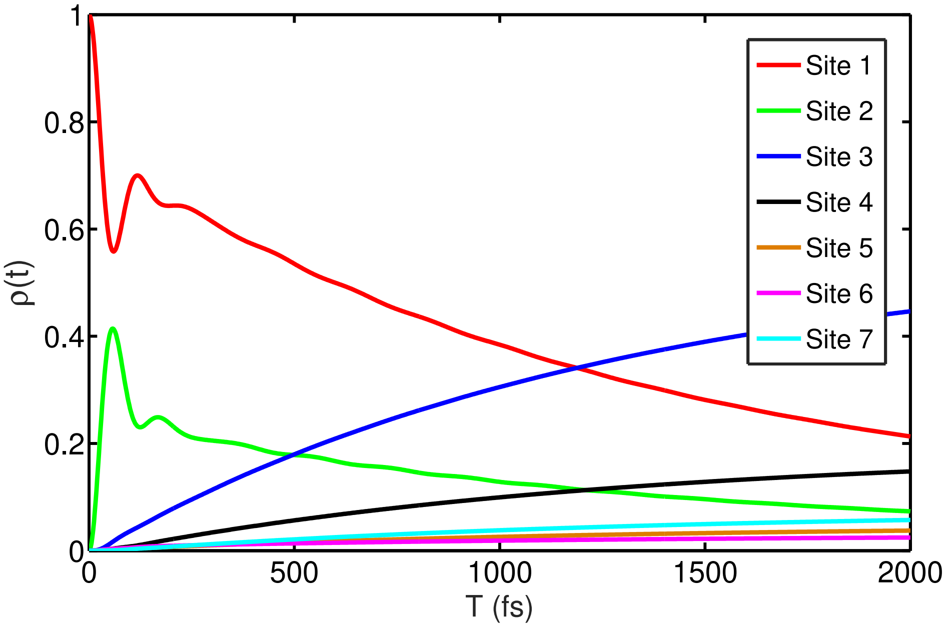

Fig. S36. Same as in Fig. S32, but for 150 K. The initial population is located on the site 1.

same form of the spectral density as described in the main text, i.e., the Ohmic part with a Drude cutoff for the electronic dephasing and the Brownian oscillator part for the vibrational relaxation.

To understand the origin of the beatings, we compare the population dynamics of two models. In the first model, the Brownian oscillator part of the spectral density consists of two vibrational modes with close-by frequencies of 180 and 200 cm^{-1} (the two-mode model). In the second model, we consider the same spectral density with only one vibrational mode of 180 cm^{-1} as used in the main text (the single-mode model). The population dynamics of two models are displayed in Fig. S38. We also retrieve the residuals by removing the kinetics with an exponential fit and show the corresponding oscillatory dynamics

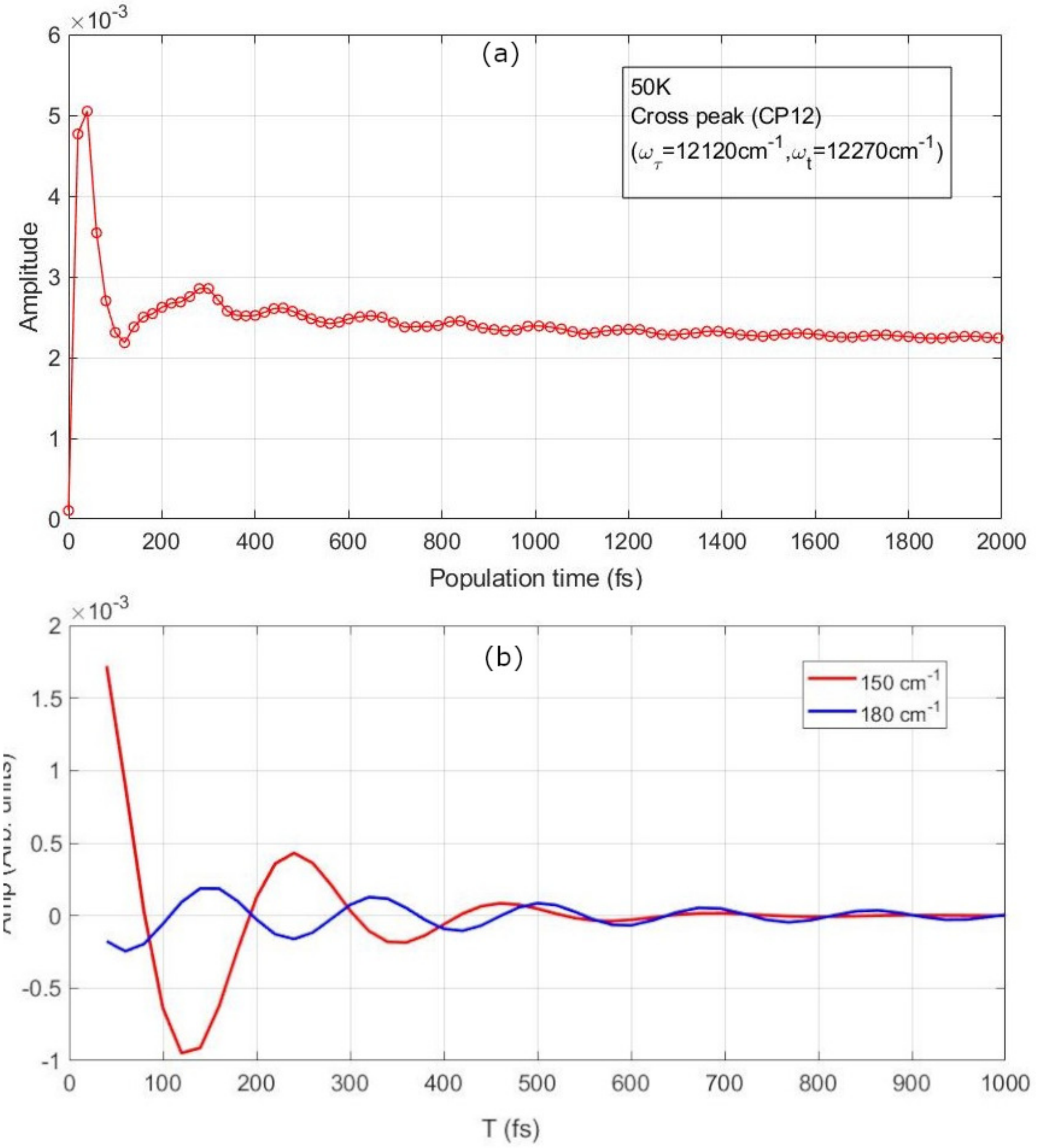

Fig. S37. (a) The time evolution of the cross peak (CP21). The time points are highlighted by red solid circles. (b) The retrieved electronic (red solid line) and vibrational (blue solid line) coherences with lifetimes of 137 fs and 420 fs, respectively.

(magnified by a factor of 5) in Fig. S38. For the two-mode model (Fig. S38(a)), one can clearly observe a two-frequency beating in the residuals, which is absent in the single-mode model (Fig. S38(b)). The simple model calculations illustrate that the observed beatings in the signal is due to vibrational modes with close-by frequencies in the electronic ground state, rather than due to the enhancement of the electronic coherence by vibronic couplings.

XVIII. Analysis of the non-Markovinity in the population transfers of the FMO complex

In this section, we study the non-Markovian dynamics of the energy transfer in the FMO complex. To this end, we calculate the population dynamics of the FMO complex and quantify the non-Markovianity by the dynamics of the trace distance between a pair of quantum states ρ_1 and ρ_2, defined as (15)

$$D(\rho_1, \rho_2) = \frac{1}{2}\mathrm{tr}|\rho_1 - \rho_2| \,, \tag{S14}$$

where $|O| = \sqrt{O^{\dagger}O}$. For the case of the FMO complex, we set the initial states as $\rho_1(0) = \rho_{11}$ and $\rho_2(0) = \rho_{66}$, i.e., the initial excitation is localized at site 1 and 6 (see Fig. 1(a) of the main text), respectively. The time evolution of the trace distance at temperatures of 50 K, 80 K, and 150 K are shown in Figs. S39(a), (b), and (c), respectively. One can readily observe that the

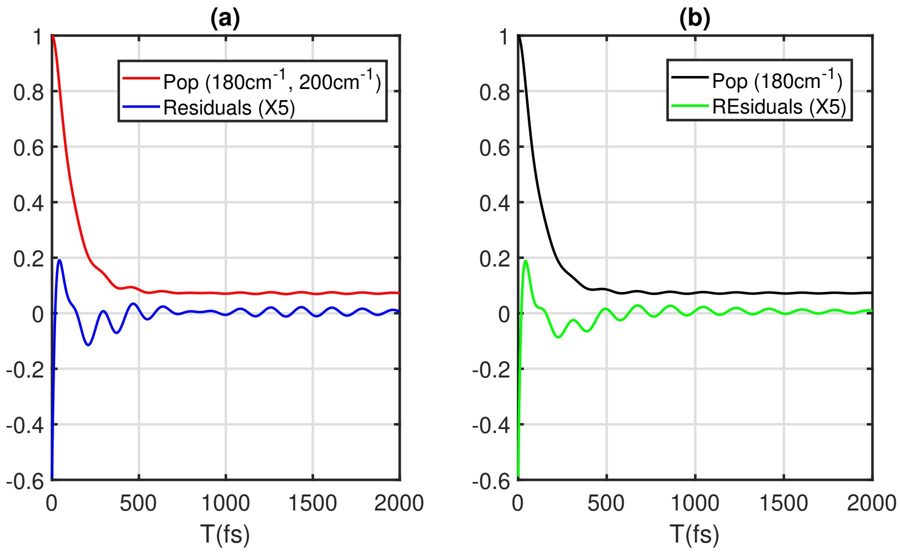

Fig. S38. (a) Population dynamics of the dimer model (took from pigment 3 and 4) and residuals are presented as red solid line and blue solid line. The beatings from two vibrations at 180 and 200 cm^{-1} are clearly shown in (a). The population dynamics of the electronic dimer model with one particular mode at 180 cm^{-1} is shown as black solid line in (b), the obtained residuals are plotted as a green solid line.

trace distance exhibits a purely monotonic decay, and the decay is faster at higher temperatures. This observation is consistent with Ref. (15). We conclude that the exciton dynamics in the FMO complex is Markovian, even at low temperature.

XIX. Time non-local quantum master equation

The Hamiltonian describing the electronic excitations of a molecular aggregate coupled to a dissipative environment can be written as

$$H = H_S + H_B + H_{SB}\,. \qquad \text{[S15]}$$

Here, H_S is the system Hamiltonian

$$H_S = \epsilon_g |g\rangle\langle g| + \sum_m^N \epsilon_m |m\rangle\langle m| + \sum_{m\neq l}^N J_{ml} |m\rangle\langle l|\,, \qquad \text{[S16]}$$

where $|g\rangle$ and $|m\rangle$ represent the ground state and the mth excited site with energy ϵ_m, respectively, and the J_{ml} are the excitonic couplings. H_B is the bath Hamiltonian

$$H_B = \sum_m^N \sum_\xi \hbar\omega_{m\xi} b^\dagger_{m\xi} b_{m\xi}\,, \qquad \text{[S17]}$$

which models the effect of the surrounding protein environment. Here, $b^\dagger_{m\xi}(b_{m\xi})$ is the creation (annihilation) operator of the ξth phonon mode associated with site m, and $\omega_{m\xi}$ is its angular frequency. H_{SB} describes interactions of the system with the phonon bath in the form

$$H_{SB} = \sum_m V_m W_m\,, \qquad \text{[S18]}$$

where we have defined $V_m = |m\rangle\langle m|$ and $W_m = -\sum_\xi c_{m\xi}(b^\dagger_{m\xi} + b_{m\xi})$. $c_{m\xi}$ is the coupling constant between the mth pigment and ξth phonon mode, and is specified by the spectral density

$$J_m(\omega) = \pi \sum_\xi \hbar^2 c^2_{m\xi} \delta(\omega - \omega_{m\xi})\,. \qquad \text{[S19]}$$

We consider one overdamped mode and one underdamped mode to model the effect of the bath. The corresponding spectral density can be expressed as

$$J(\omega) = \frac{2\Lambda\Gamma\omega}{\omega^2 + \Gamma^2} + \frac{4S\gamma_{\rm vib}\omega^3_{\rm vib}\omega}{(\omega^2 - \omega^2_{\rm vib})^2 + 4\gamma^2_{\rm vib}\omega^2}\,. \qquad \text{[S20]}$$

Here, Λ and Γ^{-1} are the damping strength and the bath relaxation time of the overdamped mode, respectively. S, $\omega_{\rm vib}$ and $\gamma^{-1}_{\rm vib}$ are the Huang-Rhys factor, the vibrational frequency and the vibrational relaxation time of the underdamped mode,

respectively. From fitting the linear absorption spectrum and 2D spectra, we find $\Lambda = 120$ cm^{-1}, $\Gamma^{-1} = 30$ fs, $S = 0.025$, $\omega_{\rm vib} = 180$ cm^{-1}, and $\gamma_{\rm vib}^{-1} = 1000$ fs.

The phonon correlation function $C_m(t)$ of the mth site can be expressed as

$$C_m(t) = a_m(t) - ib_m(t) = \int_0^\infty \frac{d\omega}{\pi} J_m(\omega)\cos(\omega t)\coth\left(\frac{\beta\omega}{2}\right) - i\int_0^\infty \frac{d\omega}{\pi} J_m(\omega)\sin(\omega t)\,, \quad \text{[S21]}$$

where $\beta = 1/k_BT$ is the inverse temperature of the environment. We evaluated Eq. (S21) analytically by using the Padé spectral decomposition (16, 17)

$$\frac{1}{1-e^{-\beta\omega}} = \frac{1}{\beta\omega} + \frac{1}{2} + \sum_{l=1}^{N_l}\left(\frac{\eta_l}{\beta\omega + i\beta\nu_l} + \frac{\eta_l}{\beta\omega - i\beta\nu_l}\right)\,. \quad \text{[S22]}$$

Here η_l and ν_l are the prefactor and frequency of the lth Padé term, respectively. For the spectral density of Eq. (S20), one obtains the analytical formula

$$\begin{aligned}
C(t) = &\quad a(t) - ib(t)\,,\\
a(t) = &\quad \Lambda\Gamma\cot\left(\frac{\beta\Gamma}{2}\right)e^{-\Gamma t} + \frac{S\omega_{\rm vib}^3}{2\widetilde{\omega}_{\rm vib}}\left[\coth\frac{\beta(\widetilde{\omega}_{\rm vib}+i\gamma_{\rm vib})}{2}e^{i(\widetilde{\omega}_{\rm vib}+i\gamma_{\rm vib})t} - \coth\frac{\beta(-\widetilde{\omega}_{\rm vib}+i\gamma_{\rm vib})}{2}e^{i(-\widetilde{\omega}_{\rm vib}+i\gamma_{\rm vib})t}\right]\\
&-\frac{4\Lambda\Gamma}{\beta}\sum_{l=1}^{N_l}\frac{\eta_l\nu_l}{\Gamma^2-\nu_l^2}e^{-\nu_l t} - \frac{8S\gamma_{\rm vib}\omega_{\rm vib}^3}{\beta}\sum_{l=1}^{N_l}\frac{\eta_l\nu_l}{[(\omega_{\rm vib}^2+\nu_l^2)^2 - 4\gamma_{\rm vib}^2\nu_l^2]}e^{-\nu_l t}\,,\\
b(t) = &\quad \Lambda\Gamma e^{-\Gamma t} - i\frac{S\omega_{\rm vib}^3}{2\widetilde{\omega}_{\rm vib}}\left[e^{i(\widetilde{\omega}_{\rm vib}+i\gamma_{\rm vib})t} - e^{i(-\widetilde{\omega}_{\rm vib}+i\gamma_{\rm vib})t}\right]\,,
\end{aligned} \quad \text{[S23]}$$

with $\widetilde{\omega}_{\rm vib} = \sqrt{\omega_{\rm vib}^2 - \gamma_{\rm vib}^2}$.

The dynamics of an open quantum system is described by the Liouville-von Neumann equation

$$\frac{\partial}{\partial t}\rho_{\rm tot}(t) = -\frac{i}{\hbar}[H, \rho_{\rm tot}(t)] \quad \text{[S24]}$$

for the full (system and bath) density matrix $\rho_{\rm tot}(t)$. The reduced density matrix

$$\rho_S(t) = {\rm Tr}_{\rm bath}[\rho_{\rm tot}(t)] \quad \text{[S25]}$$

is then obtained by taking the trace of $\rho_{\rm tot}(t)$ with respect to the bath modes. For the numerical integration of Eq. (S25), we applied the established method of the time-nonlocal quantum master equation (18–21)

$$\frac{\partial}{\partial t}\rho_S(t) = -iL_S\rho_S(t) + \int_0^t dt' K(t,t')\rho_S(t')\,, \quad \text{[S26]}$$

where

$$\begin{aligned}
K(t,t') &= \sum_m V_m^\times U_s(t,t')\left[-a(t-t')V_m^\times + ib(t-t')V_m^\circ\right]\,,\\
U_s(t,t') &= T_+\left[-i\int_{t'}^t dt'' L_S(t'')\right]\,.
\end{aligned} \quad \text{[S27]}$$

Since the correlation function in Eq. (S23) takes the form of a linear combination of exponential functions, i.e., $C(t) = a(t) - ib(t) = \sum_k (c_k^r - ic_k^i)e^{-\gamma_k t}$, we can introduce the auxiliary density matrices

$$\rho_{mk}(t) = \int_0^t dt' e^{-\gamma_k(t-t')}U_s(t,t')\left(c_k^r V_m^\times - ic_k^i V_m^\circ\right)\rho_S(t')\,. \quad \text{[S28]}$$

With these definitions, the quantum master equation in Eq. (S26) can be rewritten as

$$\begin{aligned}
\dot{\rho}_S(t) &= -iL_S\rho_S(t) - \sum_m V_m^\times\sum_k\rho_{mk}(t)\,,\\
\dot{\rho}_{mk}(t) &= \left(c_k^r V_m^\times - ic_k^i V_m^\circ\right)\rho_S(t) - (\gamma_k + iL_S)\rho_{mk}(t)\,.
\end{aligned} \quad \text{[S29]}$$

This set of coupled time non-local quantum master equations was used for the calculations of the quantum dynamics and the resulting spectra.

References

1. C Azai, et al., A heterogeneous tag-attachment to the homodimeric type 1 photosynthetic reaction center core protein in the green sulfur bacterium chlorobaculum tepidum. *Biochimica et Biophys. Acta (BBA) - Bioenerg.* **1807**, 803–812 (2011).
2. TM Wahlund, CR Woese, RW Castenholz, MT Madigan, A thermophilic green sulfur bacterium from new zealand hot springs, chlorobium tepidum sp. nov. *Arch. Microbiol.* **156**, 81–90 (1991).
3. H Oh-Oka, S Kamei, H Matsubara, M Iwaki, S Itoh, Two molecules of cytochrome c function as the electron donors to p840 in the reaction center complex isolated from a green sulfur bacterium, chlorobium tepidum. *FEBS Lett.* **365**, 30–34 (1995).
4. S Mukamel, *Principles of Nonlinear Optics and Spectroscopy.* (Oxford University Press, New York), (1995).
5. A Cohen, J Kovacevic, Wavelets: the mathematical background. *Proc. IEEE* **84**, 514–522 (1996).
6. JD Harrop, SN Taraskin, SR Elliott, Instantaneous frequency and amplitude identification using wavelets: Application to glass structure. *Phys. Rev. E* **66**, 026703 (2002).
7. JC van den Berg, *Wavelets in Physics.* (Cambridge University Press, Cambridge), (1999).
8. M Rätsep, A Freiberg, Electron–phonon and vibronic couplings in the fmo bacteriochlorophyll a antenna complex studied by difference fluorescence line narrowing. *J. Lumin.* **127**, 251–259 (2007).
9. B Hein, C Kreisbeck, T Kramer, M Rodríguez, Modelling of oscillations in two-dimensional echo-spectra of the fenna–matthews–olson complex. *New J. Phys.* **14**, 023018 (2012).
10. M Cho, HM Vaswani, T Brixner, J Stenger, GR Fleming, Exciton analysis in 2d electronic spectroscopy. *J. Phys. Chem. B* **109**, 10542–10556 (2005).
11. MF Gelin, D Egorova, W Domcke, Efficient method for the calculation of time- and frequency-resolved four-wave mixing signals and its application to photon-echo spectroscopy. *J. Chem. Phys.* **123**, 164112 (2005).
12. MF Gelin, W Domcke, Alternative view of two-dimensional spectroscopy. *J. Chem. Phys.* **144**, 194104 (2016).
13. A Ishizaki, Y Tanimura, Quantum dynamics of system strongly coupled to low-temperature colored noise bath: Reduced hierarchy equations approach. *J. Phys. Soc. Jpn.* **74**, 3131–3134 (2005).
14. M Tanaka, Y Tanimura, Quantum dissipative dynamics of electron transfer reaction system: Nonperturbative hierarchy equations approach. *J. Phys. Soc. Jpn.* **78**, 073802 (2009).
15. BH Liu, et al., Experimental control of the transition from markovian to non-markovian dynamics of open quantum systems. *Nat. Phys.* **7**, 931–934 (2011).
16. J Hu, RX Xu, Y Yan, Communication: Padéspectrum decomposition of fermi function and bose function. *J. Chem. Phys.* **133**, 101106 (2010).
17. J Hu, M Luo, F Jiang, RX Xu, Y Yan, Padéspectrum decompositions of quantum distribution functions and optimal hierarchical equations of motion construction for quantum open systems. *J. Chem. Phys.* **134**, 244106 (2011).
18. J Cao, A phase-space study of bloch–redfield theory. *J. Chem. Phys.* **107**, 3204–3209 (1997).
19. C Meier, DJ Tannor, Non-markovian evolution of the density operator in the presence of strong laser fields. *J. Chem. Phys.* **111**, 3365–3376 (1999).
20. U Kleinekathöfer, Non-markovian theories based on a decomposition of the spectral density. *J. Chem. Phys.* **121**, 2505–2514 (2004).
21. J Wu, F Liu, Y Shen, J Cao, RJ Silbey, Efficient energy transfer in light-harvesting systems, i: optimal temperature, reorganization energy and spatial–temporal correlations. *New J. Phys.* **12**, 105012 (2010).

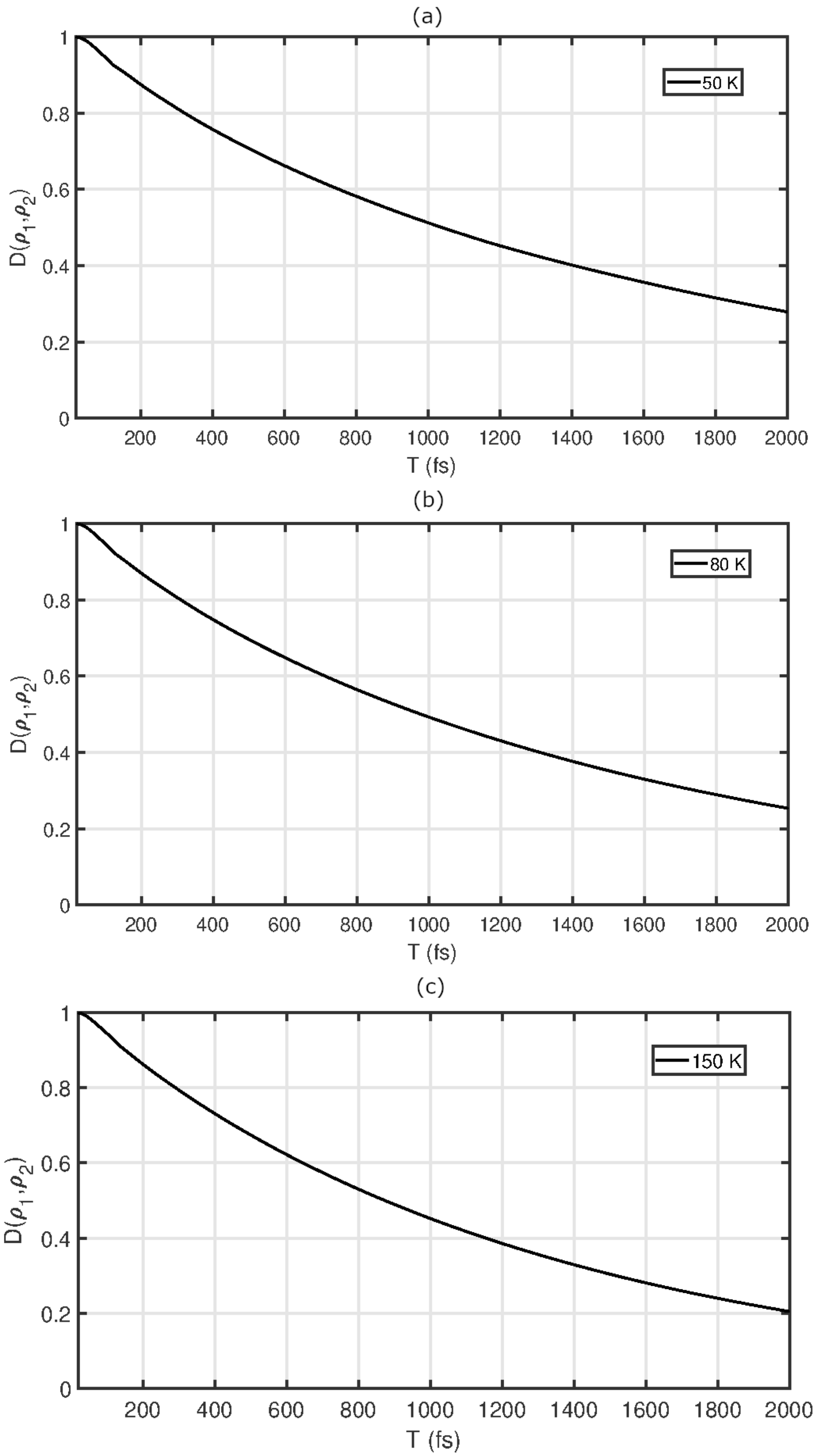

Fig. S39. Time evolution of the trace distance defined in Eq. (S14) in the FMO complex at the temperatures 50 K (a), 80 K (b), and 150 K (c).
